# Differences in neurotropism and neurotoxicity among retrograde viral tracers

**DOI:** 10.1186/s13024-019-0308-6

**Published:** 2019-02-08

**Authors:** Leqiang Sun, Yajie Tang, Keji Yan, Jinsong Yu, Yanyan Zou, Weize Xu, Ke Xiao, Zhihui Zhang, Weiming Li, Beili Wu, Zhe Hu, Kening Chen, Zhen F. Fu, Jinxia Dai, Gang Cao

**Affiliations:** 1 State Key Laboratory of Agricultural Microbiology, Huazhong Agricultural University, Wuhan, 430070 China; 2 Bio-Medical Center, Huazhong Agricultural University, Wuhan, 430070 China; 3 College of Veterinary Medicine, Huazhong Agricultural University, Wuhan, 430070 China; 4 Key Laboratory of Preventive Veterinary Medicine in Hubei Province, Wuhan, 430070 China; 5 College of Informatics, Huazhong Agricultural University, Wuhan, 430070 China; 6 College of Life Science and Technology, Huazhong Agricultural University, Wuhan, 430070 China; 7 Departments of Pathology, College of Veterinary Medicine, University of Georgia, Athens, GA 30602 USA

**Keywords:** Neurotropic Virus, Retrograde tracing, pseudorabies virus (PRV), rabies virus (RV), rAAV2-retro, Neurotropism, Neurotoxicity, Multi-trans-synaptic Tracing, Mono-synaptic Tracing, RNA Sequencing

## Abstract

**Background:**

Neurotropic virus-based tracers have been extensively applied in mapping and manipulation of neural circuits. However, their neurotropic and neurotoxic properties remain to be fully characterized.

**Methods:**

Through neural circuit tracing, we systematically compared the neurotropism discrepancy among different multi-trans-synaptic and mono-synaptic retrograde viral tracers including pseudorabies virus (PRV), rabies virus (RV), and the newly engineered retro adeno-associated virus (rAAV2-retro) tracers. The (single-cell) RNA sequencing analysis was utilized for seeking possible attribution to neurotropism discrepancy and comparing cell toxicity caused by viral infection between glycoprotein-deleted RV (RV-∆G) and rAAV2-retro. Viral toxicity induced microglia activation and neuronal protein change were evaluated by immunohistochemistry.

**Results:**

Multi-trans-synaptic retrograde viral tracers, PRV and RV, exhibit differential neurotropism when they were used for central neural circuit tracing from popliteal lymph nodes. Mono-synaptic retrograde tracers, including RV-∆G and rAAV2-retro, displayed discrepant neurotropic property, when they were applied to trace the inputs of lateral hypothalamic area and medial preoptic nucleus. rAAV2-retro demonstrated preference in cerebral cortex, whereas RV-∆G prefers to label basal ganglia and hypothalamus. Remarkably, we detected a distinct preference for specific cortical layer of rAAV2-retro in layer 5 and RV-∆G in layer 6 when they were injected into dorsal lateral geniculate nucleus to label corticothalamic neurons in primary visual cortex. Complementation of TVA receptor gene in RV-resistant neurons enabled EnvA-pseudotyped RV infection, supporting receptors attribution to viral neurotropism. Furthermore, both RV-∆G and rAAV2-retro exerted neurotoxic influence at the injection sites and retrogradely labeled sites, while the changes were more profound for RV-∆G infection. Finally, we demonstrated a proof-of-concept strategy for more comprehensive high-order circuit tracing of a specific target nucleus by combining rAAV2-retro, RV, and rAAV tracers.

**Conclusions:**

Different multi-trans-synaptic and mono-synaptic retrograde viral tracers exhibited discrepant neurotropism within certain brain regions, even cortical layer preference. More neurotoxicity was observed under RV-∆G infection as compared with rAAV2-retro. By combining rAAV2-retro, RV, and rAAV tracers, high-order circuit tracing can be achieved. Our findings provide important reference for appropriate application of viral tracers to delineate the landscape and dissect the function of neural network.

**Electronic supplementary material:**

The online version of this article (10.1186/s13024-019-0308-6) contains supplementary material, which is available to authorized users.

## Background

The human brain is a highly complex network in which tremendous numbers (~10^11^) of neurons with diverse cell types are connected to form approximately 10^14-15^ synapses. Such sophisticated organization of neural circuits establish the structural foundation underlying brain functions such as cognition, emotion, memory, sensation, and movement. Delineating the fine architecture and function of neural circuits is pivotal for understanding complex brain functions, and is largely dependent on the availability of effective tracing and manipulation techniques.

In early neural circuit studies, several tracing dyes were applied to label presynaptic and postsynaptic neurons, which helps to resolve mesoscopic connections between different brain nuclei [1–3]. Horseradish peroxidase (HRP) was the first tracer used for circuit mapping [4], followed by a series of anterograde or retrograde tracers including carbocyanine dyes [5, 6], FluoroGold [7–9], cholera toxin [10–12], and fluorescent microspheres [13, 14]. However, these conventional neural tracers lack selectivity, bear little-to-no trans-synaptic ability, and cannot be used to deliver exogenous genes [2, 15].

Currently, neurotropic virus-based tracers are most widely utilized for identifying presynaptic or postsynaptic neurons, higher order neural connections, and neural circuit functions [15–22]. Different viruses such as adeno-associated virus (AAV) [23–29, 30, 31], rabies virus (RV) [16, 17, 21, 32], pseudorabies virus (PRV) [18, 33–35], canine adenovirus (CAV) [36–39], and Herpes simplex virus (HSV) [40, 41] have been engineered to trace and manipulate neural circuits. Notably, the development of mono-trans-synaptic tracing tools has enabled the investigation of direct upstream connections within certain neural circuits [21, 42–46]. Recently, an engineered AAV variant rAAV2-retro was examined to bear retrograde tracing potential, although its mechanism of action remains poorly understood [29].

Relative to conventional tracers, neurotropic viruses produce a stronger signal due to viral genome replication and exogenous gene expression in the recipient neuron [21, 29, 32, 47]. Moreover, these tracing tools can selectively label specific neurons with the aid of a specific promoter or Cre/lox techniques [21, 39, 48]. In addition, neurotropic viruses expressing optogenetic, chemogenetic, and Ca^2+^/voltage-sensitive genes can be used to monitor and manipulate activity within specific neural circuits [49–52]. Meanwhile, previous studies have indicated that both natural and engineered neurotropic viruses exhibit a preference with regard to the direction of labeling. Herpes Simplex virus HSV129 strain transport mainly in anterograde direction [40, 53]. Vesicular stomatitis virus (VSV) glycoprotein-enveloped RV has also been used as an anterograde tracer [54]. While RV [16, 55, 56], CAV [36], PRV lacking the gE and gI gene [15, 57, 58], and rAAV2-retro virus [29] demonstrate a significant preference for retrograde labeling. Given these advantages, neurotropic virus-based tracing tools are highly suitable for mapping fine neural circuits and their functions.

Although these anterograde and retrograde neurotropic viruses greatly facilitate resolving neural circuit connection and function, it is of noteworthy that different tracing viruses may cause discrepant labeling results. That is, while some circuits can be equally traced by different viruses, selective or preferential tracing for other circuits will exist for different viruses. It has been reported that HSV1 and PRV can infect neurons including primary sensory neurons, motoneurons, sympathetic and parasympathetic neurons innervating the injection sites [15, 59, 60]. However, both viruses have a greater affinity for small primary sensory neurons and autonomic neurons than motorneurons [15, 61, 62]. As the autonomic and sensory neurons innervating the muscle are rarely infected by RV strain CVS-11 when injected into the muscles of primates, rats and guinea pigs, this virus was proposed as a specific tracer for motoneuron network in this animals [15, 63, 64]. Albisetti et al. identified two classes of sensory neurons that are resistant to direct and trans-synaptic RV infection from the spinal cord, possibly due to impaired virus adsorption [65]. Interestingly, it has been reported that newly engineered CVS-N2c∆G and RV with codon-optimized chimeric glycoprotein (G) consisting of G from B19G RV strain and Pasteur RV strain can increase the tracing efficiency, indicating that even different strains of the RV may have different neurotropism [45, 51]. Moreover, when CAV2-Cre and HSV1 were applied for retrogradely tracing from medial prefrontal cortex to basolateral amygdala, they exhibited different tropism in the basolateral amygdala [66]. Such distinct neurotropism is most likely attributable to the expression of distinct receptors required for neurotropic virus invasion in different neurons [67]. However, the receptors for different tracing viruses remain to be fully characterized. Although different viral tracers are widely used in neural circuit study, few studies have systematically compared the tracing characteristics of different neurotropic viruses, greatly hampering its proper application in elucidating neural circuits.

Furthermore, several viral tracers are reported to cause cytotoxicity of the infected neuron during circuit tracing [15]. HSV1 and PRV induce rapid neuronal degeneration of the infected neurons [15, 19]. For glycoprotein-deletion rabies virus, although the cytopathic change is negligible in the early infection stage, it will cause altered electrophysiological properties and even neuron death around 16 days post injection [32, 68]. However, systematic comparison analysis of neurotoxicity induced by different circuit tracers has not been well documented.

Therefore, in the present study, we aimed to systematically compare the neurotropic discrepancies of different retrograde tracing viruses that are widely used in current neuroscience research, including PRV, RV, and the newly engineered rAAV2-retro virus. We further aimed to elucidate the mechanisms underlying neurotropic discrepancies of these tracing viruses by using EnvA-TVA complementation system and analyzing gene expression profiles in different groups of virus-labeled neurons via single-cell RNA sequencing (RNA-seq). Then the neurotoxicity of RV-∆G and rAAV2-retro virus tracers was compared via RNA-seq and immunohistochemistry. Finally, we demonstrated a proof-of-concept strategy for higher-order circuit tracing by combining different viral tracers. Our findings may represent an important reference for proper application of neurotropic viruses in the comprehensive investigation of neural circuit architecture and function.

## Results

### PRV and RV Exhibit Differences in Neurotropism during Multi-trans-synaptic Retrograde Tracing

**Fig. 1:**
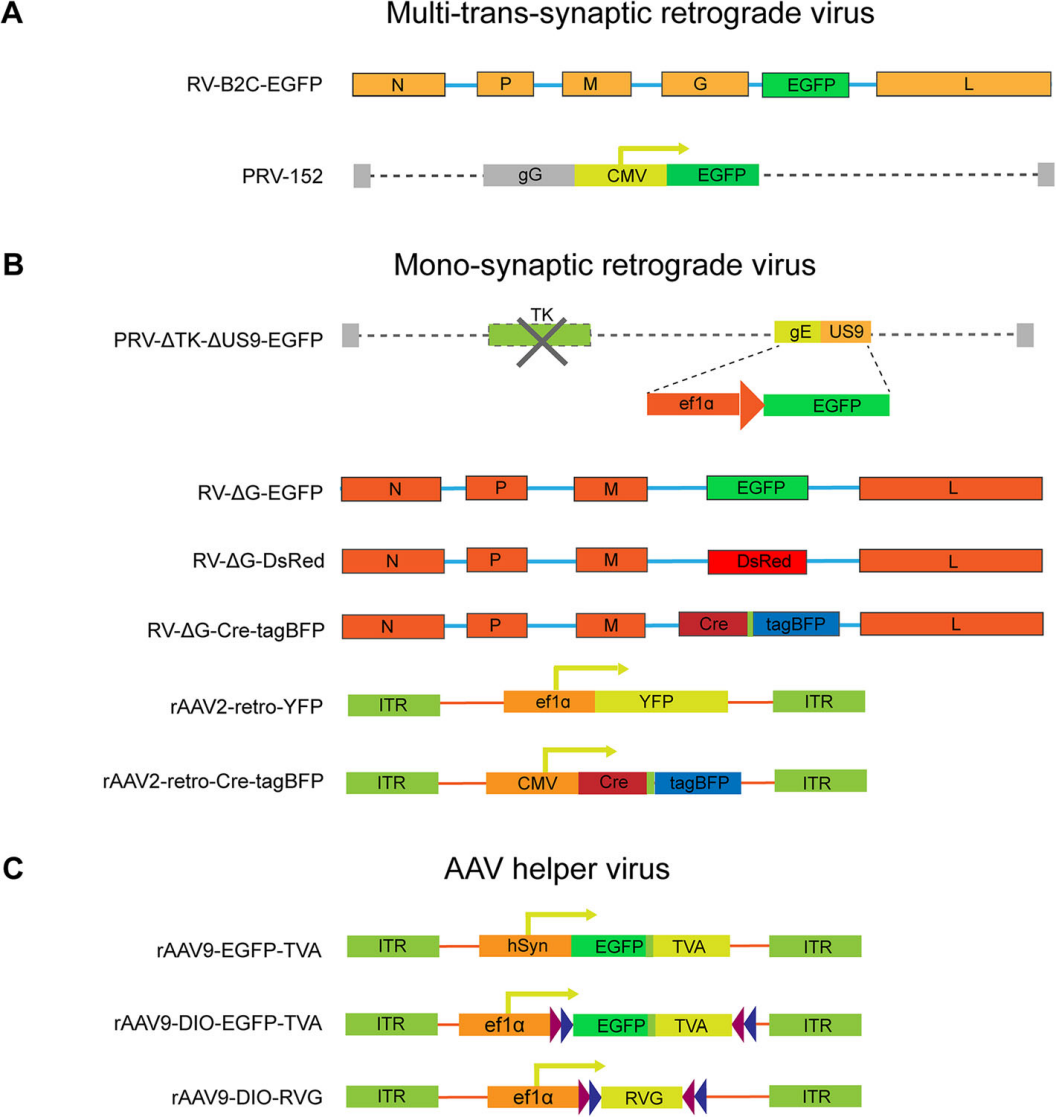
The structures of all viral tracers used in our study. (**a**) Structures of multi-trans-synaptic retrograde viruses including RV-B2C-EGFP and PRV-152 are illustrated. For RV-B2C-EGFP, EGFP gene was inserted between G- and L-coding sequences of CVS-B2C strain. For PRV-152, EGFP was driven by the CMV promoter following gG gene of the Bartha strain. (**b**) Structures of mono-synaptic retrograde viruses are illustrated. Because TK (thymidine kinase) gene is involved in viral replication and US9 is responsible for anterograde transport, a retrograde mono-synaptic tracing virus PRV-∆TK-∆US9-EGFP was obtained by deleting both TK and US9 genes, in which the location of US9 gene was replaced by CMV promoter and EGFP gene via homologous recombination on the BAC backbone of the Becker strain. For the RV-∆G mono-synaptic virus, EGFP, DsRed and Cre-T2A-tagBFP was respectively inserted in the rabies virus genome to replace the G gene. For the rAAV2-retro, the ef1a and CMV promotor was used to drive YFP and Cre-T2A-tagBFP expression, respectively. (**c**) Structures of AAV helper viruses. The hSyn and ef1a promoter were used to drive TVA, DIO-TVA and DIO-RVG elements

Pseudorabies virus (PRV) is the most commonly used virus for multi-trans-synaptic tracing (trans-multiple synapse) of central neural circuits from peripheral innervating targets. In the present study, we compared the neurotropic actions of a fixed RV derivative RV-B2C-EGFP (EGFP are inserted between G- and L-coding sequences of RV-B2C strain) with those of the PRV-Bartha derivative PRV-152 (Fig. 1a). As popliteal lymph node is anatomically isolated and with convenient size for precise virus injection, it is selected for neurotropism comparison among multi-trans-synaptic retrograde tracing viruses. These GFP expressing viruses were injected into mouse left popliteal lymph node to trace the upstream multi-trans-synaptic circuits in the central nervous system, respectively.

**Table 1:** Abbreviation for brain structures

abbreviation	brain structure	abbreviation	brain structure
2Cb	2nd Cerebellar lobule	LSI	lateral septal nucleus, intermediate part
AAD	anterior amygdaloid area, dorsal part	M1	primary motor cortex
AAV	anterior amygdaloid area, ventral part	M2	secondary motor cortex
ACo	anterior cortical amygdaloid nucleus	MDM	mediodorsal thalamic nucleus, medial part
ADP	anterodorsal preoptic nucleus	MeA	medial amygdaloid nucleus, anterior part
AHA	anterior hypothalamic area, anterior part	MePD	medial amygdaloid nucleus, posterodorsal part
AHC	anterior hypothalamic area, central part	MePV	medial amygdaloid nucleus, posteroventral part
AHiPM	amygdalohippocampal area, posteromedial part	MnPO	median preoptic nucleus
AHiAL	amygdalohippocampal area, anterolateral part	MnR	median raphe nucleus
AHP	anterior hypothalamic area, posterior part	MO	medial orbital cortex
AI	agranular insular cortex	MPA	medial preoptic area
AID	agranular insular cortex, dorsal part	MPO	medial preoptic nucleus
AIP	agranular insular cortex, posterior part	MPtA	medial parietal association cortex
AIV	agranular insular cortex, ventral part	MS	medial septal nucleus
AO	anterior olfactory	MTu	medial tuberal nucleus
AM	anteromedial thalamic nucleus	NAc	accumbens nucleus
APir	amygdalopiriform transition area	PAG	periaqueductal gray
Arc	arcuate hypothalamic nucleus	PH	posterior hypothalamic area
Au1	primary auditory cortex	Pir	piriform cortex
AuD	secondary auditory cortex, dorsal area	PLCo	posterolateral cortical amygdaloid nucleus(C2)
AuV	secondary auditory cortex, ventral area	PMCo	posteromedial cortical amygdaloid nucleus(C3)
AVPe	anteroventral periventricular nucleus	PMD	premammillary nucleus, dorsal part
Bar	Barrington’s nucleus	PMV	premammillary nucleus, ventral part
BLA	basolateral amygdaloid nucleus anterior part	PnO	pontine reticular nucleus, oral part
BLP	basolateral amygdaloid nucleus posterior part	PPTg	pedunculopontine tegmental nucleus
BLV	basolateral amygdaloid nucleus, ventral part	PRh	perirhinal cortex
BMA	basomedial amygdaloid nucleus anterior part	PrL	prelimbic cortex
BMP	basomedial amygdaloid nucleus, posterior part	PS	parastrial nucleus
BNST	bed nucleus of the stria terminalis	PT	Paratenial thalamic nucleus
CA1	field CA1 of hippocampus	PtA	parietal association cortex
CeC	central amygdaloid nucleus, capsular part	PV	paraventricular thalamic nucleus
CeM	central amygdaloid nucleus, medial division	PVA	paraventricular thalamic nucleus, anterior part
Cg1	cingulate cortex, area 1	PVN	paraventricular hypothalamic nucleus
Cg2	cingulate cortex, area 2	RC	raphe cap
CM	central medial thalamic nucleus	RPC	red nucleus, parvicellular part
CPu	striatum	RRF	retrorubral field
CxA	cortex-amygdala transition zone	RSA	retrosplenial agranular cortex
dLGN	dorsal lateral geniculate nucleus	RSG	retrosplenial granular cortex
DEn	dorsal endopiriform nucleus	Re	reuniens thalamic nucleus
DI	dysgranular insular cortex	Rt	reticular thalamic nucleus
DM	dorsomedial hypothalamic nucleus	S1	primary somatosensory cortex
DP	dorsal peduncular cortex	S2	secondary somatosensory cortex
		SCh	suprachiasmatic nucleus
DpMe	deep mesencephalic nucleus	SFO	subfornical organ
DR	dorsal raphe nucleus	SHi	septohippocampal nucleus
ECIC	external cortex of the inferior colliculus	SI	substantia innominata
Ect	ectorhinal cortex	SNC	substantia nigra, compact part
GI	glomerular layer of the olfactory bulb	SON	supraoptic nucleus
Gi	gigantocellular reticular nucleus	Su3	supraoculomotor periaquductal gray
HDB	nucleus of the horizontal limb of the diagonal band	Su3C	Supraoculomotor cap
IL	infralimbic cortex	SubCD	subcoeruleus nucleus, dorsal part
IMD	intermediodorsal thalamic nucleus	SubCV	subcoeruleus nucleus, ventral part
IPACL	interstitial nucleus of the posterior limb of the anterior commissure, lateral part	SubI	subincertal nucleus
IPACM	interstitial nucleus of the posterior limb of the anterior commissure, medial part	TC	tuber cinereum area
LA	lateroanterior hypothalamic nucleus	TeA	temporal association cortex
LC	locus coeruleus	VDB	nucleus of the vertical limb of the diagonal band
LDTg	laterodorsal tegmental nucleus	VLPO	ventrolateral preoptic nucleus
LEnt	lateral entorhinal cortex	VMH	ventromedial hypothalamic nucleus
LGP	lateral globus pallidus	VMPO	ventromedial preoptic nucleus
LHA	lateral hypothalamic area	VO	ventral orbital cortex
LO	lateral orbital cortex	VOLT	vascular organ of the lamina terminalis
LPAG	lateral periaqueductal gray	VP	ventral pallidum
LPBC	lateral parabrachial nucleus, central part	VPM	ventral posteromedial thalamic nucleus
LPO	lateral preoptic area	VTA	ventral tegmental area
LPtA	lateral parietal association cortex	Xi	xiphoid thalamic nucleus
LSD	lateral septal nucleus, dorsal part	ZI	zona incerta

**Fig. 2:**
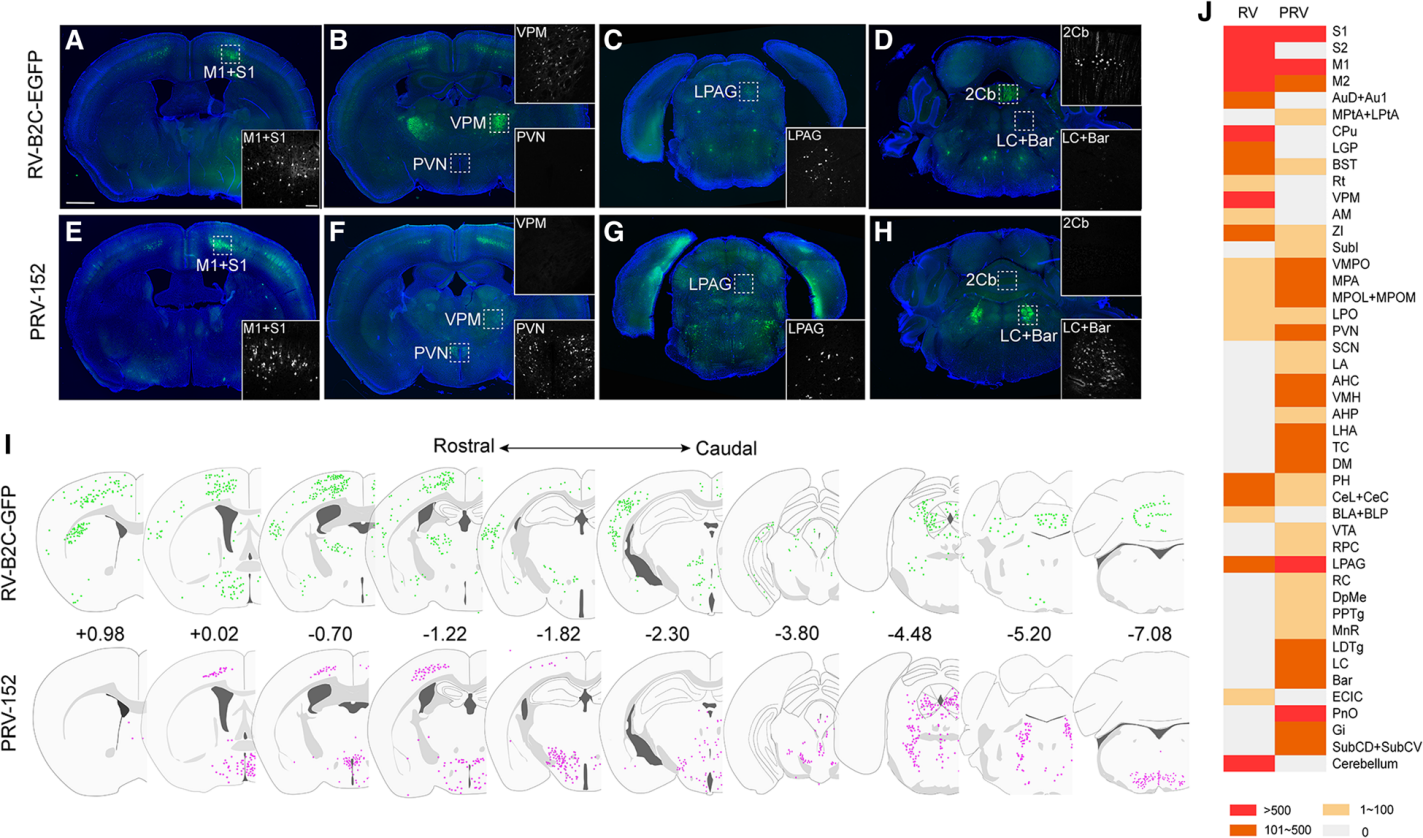
Multi-trans-synaptic retrograde tracing via injection of RV-B2C-EGFP and PRV-152 into the lymph nodes. (**a-d**) RV-B2C-EGFP retrogradely labeled M1 (A), S1 (A), VPM (B), LPAG (C), and cerebellum (D), but not PVN, LC, or Bar when injected into the lymph nodes. (**e-h**) Retrograde labeling by PRV-152 from the lymph nodes was observed in M1 (E), S1 (E), PVN (F), LPAG (G), LC and Bar (H), but not VPM (F) and cerebellum (H). (**i**) The Map of inputs to LHA labeled by RV-B2C-EGFP (upper line) and PRV-152 (lower line) at coronal view are arranged along the rostrocaudal direction. Retrogradely labeled neurons are indicated as colored dots. Numbers indicate position of sections relative to bregma (mm). (**j**) The semi-quantified labeling discrepancy between RV-B2C-EGFP and PRV-152 in the whole brain. Three mice were analyzed for each viral tracer. Insets show magnified images of the boxed regions. Scale bars = 1 mm in lower magnification images; 100 μm in higher magnification insets

Several brain regions including the primary motor cortex (M1), primary somatosensory cortex (S1), lateral periaqueductal gray (LPAG) (Fig. 2a, c, e, g), preoptic area (VMPO, MPA, MPOL+MPOM, LPO), and bed nucleus of the stria terminalis (BNST) (Fig. 2j) were labeled by both viral tracers. However, some brain regions were labeled exclusively or preferentially by either PRV-152 or RV-B2C-EGFP. Retrograde labeling of the ventral posteromedial thalamic nucleus (VPM), cerebellum (Fig. 2b and d), striatum (CPu), and secondary somatosensory cortex (S2) (Fig. 2j) was observed when RV-B2C-EGFP was injected into the popliteal lymph node, but not when PRV-152 was injected. In contrast, numerous neurons in the paraventricular nucleus (PVN), locus coeruleus (LC), Barrington’s nucleus (Bar) (Fig. 2f and h), pontine reticular nucleus (PnO), and gigantocellular reticular nucleus (Gi) (Fig. 2j) were mostly labeled by PRV-152. The whole-brain maps of retrogradly labeled neurons (in color dots) by PRV and RV at coronal view are summarized along the rostral-to-caudal direction (Fig. 2i). Further, the semi-quantified labeling discrepancy between the two viruses along the whole brain is presented in Fig. 2j. Full names of abbreviation for brain structures are listed in Table 1. Taken together, these findings suggest that PRV and RV, which belong to different genus of viruses and contain different viral structures, exhibit differential neurotropic actions when injected into the popliteal lymph node.

**Table 2:** Information for virus injection

Virus	Volume (nL)	Titer (particles/mL)	Injection site	Coordinates (AP, ML, DV)
RV-B2C-EGFP	2000	4x10^7^	popliteal lymph node	NA
RV-152	2000	5×10^9^	popliteal lymph node	NA
PRV-∆TK-∆US9-EGFP	200	1×10^9^	LHA	(-1.0, 1.22, 4.93)
RV-∆G-EGFP	100	4×10^9^	LHA	(-1.0, 1.22, 4.93)
			dLGN	(-2.35, 2.26, 2.7)
RV-∆G-Cre-tagBFP	100	2×10^9^	LHA	(-1.0, 1.22, 4.93)
rAAV2-retro-YFP	200	2×10^12^	LHA	(-1.0, 1.22, 4.93)
			dLGN	(-2.35, 2.26, 2.7)
rAAV2-retro-Cre-tagBFP	50	8×10^12^	LHA	(-1.0, 1.22, 4.93)
			dLGN	(-2.35, 2.26, 2.7)
Virus co-injection:			LHA	(-1.0, 1.22, 4.93)
RV-∆G-EGFP	150	4×10^9^	dLGN	(-2.35, 2.26, 2.7)
rAAV2-retro-Cre-tagBFP	50	8×10^12^	MPO	(0.15, 0.5, 4.7)
EnvA-RV-∆G-DsRed	250	3×10^9^	LHA	(-1.0, 1.22, 4.93)
			mPFC	(1.6, 0.45, 0.8-1.2)
			dLGN	(-2.35, 2.26, 2.7)
rAAV9-EGFP-TVA	300	6×10^12^	mPFC	(1.6, 0.45, 0.8-1.2)
			V1	(-3.4, 2.25, 0.6)
rAAV9-DIO-G	200	6×10^12^	PVN	(-0.7, 0.25, 4.7)

### Discrepancies in Circuit Labeling with Different Mono-synaptic Retrograde Tracing Viruses

The observed differences in neurotropism between PRV and RV suggest that mono-synaptic retrograde tracers (only labeling direct input neuron from axon terminal targeting to the injection site) engineered from PRV or RV and other commonly used alternatives will result in discrepant tracing circuits. To examine this hypothesis, we compared the neural circuits identified by different mono-synaptic retrograde tracers, including glycoprotein-deleted RV (RV-∆G) derived from the SAD-B19 vaccine strain, TK (Thymidine kinase) gene-deleted PRV (PRV-∆TK-∆US9-EGFP), retro adeno-associated virus (rAAV2-retro), and retrobeads (Fig. 1b). As the fluorescent signal was hardly detectable when PRV-∆TK-∆US9-EGFP was used for mono-synaptic retrograde tracing, possibly due to the TK gene deletion caused replication deficit, we excluded it for further analysis.

**Fig. 3:**
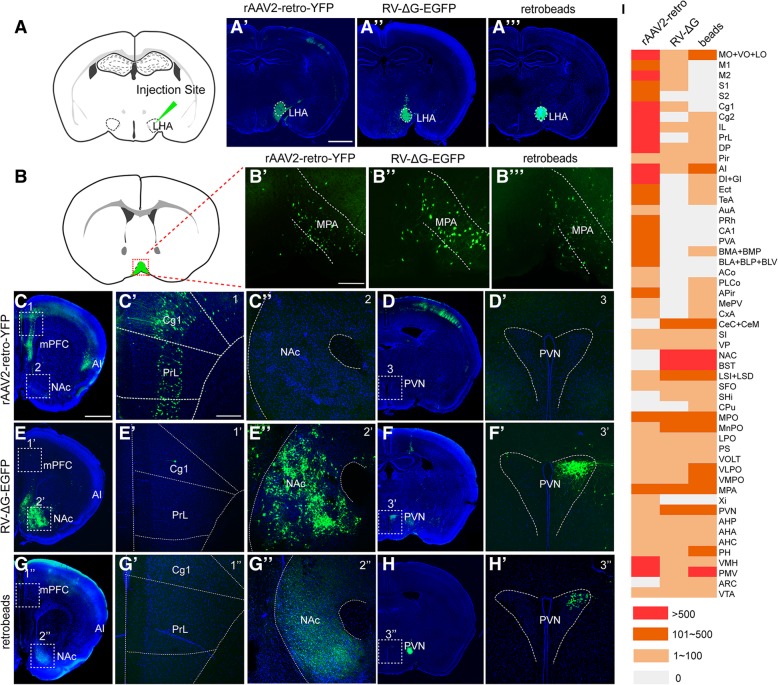
Mono-synaptic retrograde labeling of LHA input circuits using rAAV2-retro-YFP, RV-∆G-EGFP, and green retrobeads. (**a**) Schematic diagram of tracer injection into the lateral hypothalamic area (LHA). (**a’-a”’**) The injection sites of rAAV2-retro-YFP, RV-∆G-EGFP, and green retrobeads were confirmed to be localized in the LHA. (**b-b‴**) Retrograde labeling of the MPA was observed for all three tracers. (**c-d′**) Retrogradely labeled neurons were observed in the mPFC following injection of rAAV2-retro-YFP (C, C′), while no such labeling was observed in the NAc (C, C”) or PVN (D, D’). (**e-f’**) Neurons retrogradely labeled with RV-∆G-EGFP were localized in the NAc (E, E”) and PVN (F, F’), but were barely observed in the mPFC (E, E’). (**g-h′**) Green retrobeads retrogradely labeled the NAc (G, G”) and PVN (H, H’), but not the mPFC (G, G’). (**i**) The number of labeled neurons in each upstream region of the LHA was compared among the three tracers. Three mice were analyzed for each viral tracer or retrobeads. Scale bars = 1 mm for A′-A‴, C, D, E, F, G, H; 200 μm for the magnified images

We injected rAAV2-retro-YFP, RV-∆G-EGFP, and green retrobeads into the lateral hypothalamic area (LHA) (dashed line circled region in Fig. 3a-a‴), following comparing the direct upstream circuits of the LHA among the three tracers. Retrograde labeling of the medial preoptic area (MPA) (Fig. 3b-b‴) was observed for all three tracers. Fluorescence-labeled neurons in the medial prefrontal cortex (mPFC), agranular insular cortex (AI) (Fig. 3c-c′), CA1 of the hippocampus, basolateral amygdaloid nucleus (BLA+BLP+BLV), and basomedial amygdaloid nucleus (BMA+BMP) (Fig. 3i) were observed via rAAV2-retro-YFP tracing, whereas few positive neurons were detected in these areas using RV-∆G-EGFP (Fig. .3e-e′, 3i). In contrast, the nucleus accumbens (NAc), paraventricular nucleus (PVN) (Fig. .3e″-f′), central amygdaloid nucleus (CeC+CeM), and bed nucleus of the stria terminalis (BNST) (Fig. 3i) were labeled by RV-∆G-EGFP, but were barely labeled by rAAV2-retro-YFP (Fig. 3c″-d′, 3i). Retrograde labeling of the NAc and PVN was also observed when green retrobeads were used (Fig. 3g-h'). The numbers of labeled neurons in each traced region were semi-quantified and compared among the three tracers (Fig. 3i). Full names of abbreviation for brain structures are listed in Table 1.

**Fig. 4:**
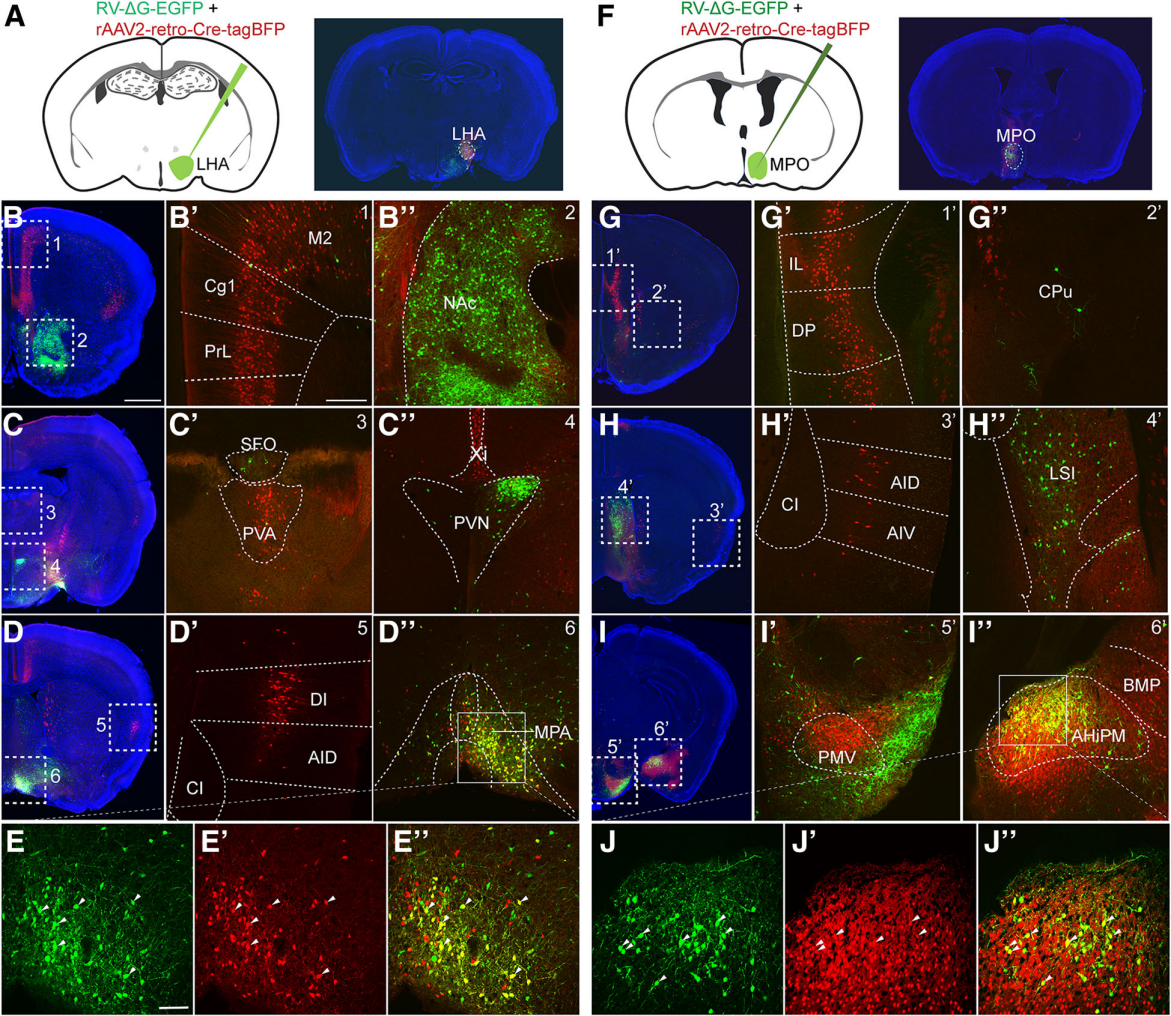
Co-injection of rAAV2-retro-Cre-tagBFP and RV-∆G-EGFP into LHA and MPO nuclei to trace input neurons. (**a**) Schematic diagram of virus co-injection into the LHA of Ai9 reporter mice and the injection sites confirmation. (**b-e”**) When the LHA was simultaneously injected with rAAV2-retro-Cre-tagBFP and RV-∆G-EGFP, discrepancies in the input circuits retrogradely labeled by the two viruses were observed. The mPFC (Cg1+PrL), M2, PVA, and DI were exclusively labeled by rAAV2-retro-Cre-tagBFP, while the NAc and PVN were preferentially labeled by RV-∆G-EGFP, although overlapping labeling was observed in the MPA. (**f**) Schematic diagram and the injection sites of virus co-injection in the medial preoptic nucleus (MPO). (**g-j”**) Injection of rAAV2-retro-Cre-tagBFP in MPO was associated with preferential labeling in the mPFC, AI and PMV. In contrast, injection of RV-∆G-EGFP in MPO was associated with preferential labeling in the LSI. Overlapping signals were observed in the AHiPM. Red, tdTomato expressed by rAAV2-retro-Cre-tagBFP-labeled cells; green, GFP expressed by RV-∆G-EGFP-labeled cells; blue, DAPI. Scale bars = 1 mm for A-D and F-I; 200 μm for B’-D’, G’-I’, B”-D”and G”-I”; 100 μm for E-E” and J-J”

Since the brightness of YFP expressed in rAAV2-retro-YFP-infected neurons was weaker than that of GFP expressed in RV-∆G-EGFP-infected neurons (Fig. 3), the presence of neurons positive for RV-∆G-EGFP but negative for rAAV2-retro-YFP may be caused by the lower YFP expression in regions upstream from the LHA. To exclude this possibility, we injected rAAV2-retro-Cre-tagBFP and RV-∆G-Cre-tagBFP viruses into the LHA of Ai9 reporter mice (Additional file 1: Figure S1A-S1C), in which even low Cre recombinase expression can trigger strong and stable tdTomato expression in the infected neurons via the Cre/LoxP system. Although rAAV2-retro-Cre-tagBFP-mediated tdTomato expression can indeed improve labeling efficiency in rAAV2-retro-YFP-negative brain regions including the NAc and PVN relative to that observed using rAAV2-retro-YFP (Fig. 3c-d', Additional file 1: Figure S1D-S1E'), the number of tdTomato-positive neurons was still much lower than that labeled by RV-∆G in these regions (Fig. 3e-f′, Additional file 1: Figure S1D-S1E', Additional file 1: Figure S1F-S1G'). Furthermore, we observed no significant difference between RV-∆G-Cre-tagBFP and RV-∆G-EGFP tracing results, likely due to the high level of gene expression in the RV system (Fig. 3e-f' and Additional file 1: Figure S1F-S1G'). Using this Cre/LoxP system, we still observed significant differences between the circuits traced by the RV-∆G-Cre-tagBFP and rAAV2-retro-Cre-tagBFP viruses (Additional file 1: Figure S1D-S1G'), which are consistent with the labeling pattern that observed using rAAV2-retro-YFP and RV-∆G-EGFP. For example, strong labeling was observed in the mPFC when rAAV2-retro-Cre-tagBFP was used, and in the NAc and PVN when RV-∆G-Cre-tagBFP was used. To further exclude the possibility that the discrepancies in neurotropism were caused by differences in injection deviation, we injected a mixture of rAAV2-retro-Cre-tagBFP and RV-∆G-EGFP into the LHA (Fig. 4a) of Ai9 reporter mice. Under this protocol, we also obtained consistent results with that when two viruses were injected separately (Fig. 3, Additional file 1: Figure S1, and Fig. 4b-e”).

To determine whether this discrepancy occurred only within the circuits upstream from the LHA, we also injected these tracers into the medial preoptic nucleus (MPO). Similarly, a clear discrepancy in the labeled circuits from MPO between two viruses was observed. As shown in Figure 4f-j”, mPFC and premammillary nucleus ventral part (PMV) were intensively labeled by rAAV2-retro-Cre-tagBFP only, whereas lateral septal nucleus intermediate part (LSI) were predominately labeled by RV-∆G-EGFP. Notably, both viruses labeled the medial preoptic area when injected into the LHA, as well as the amygdala when injected into the MPO, in which the labeled neurons exhibited large overlap with one another (Fig. 4d-e” and Fig. 4i-j”).

**Fig. 5:**
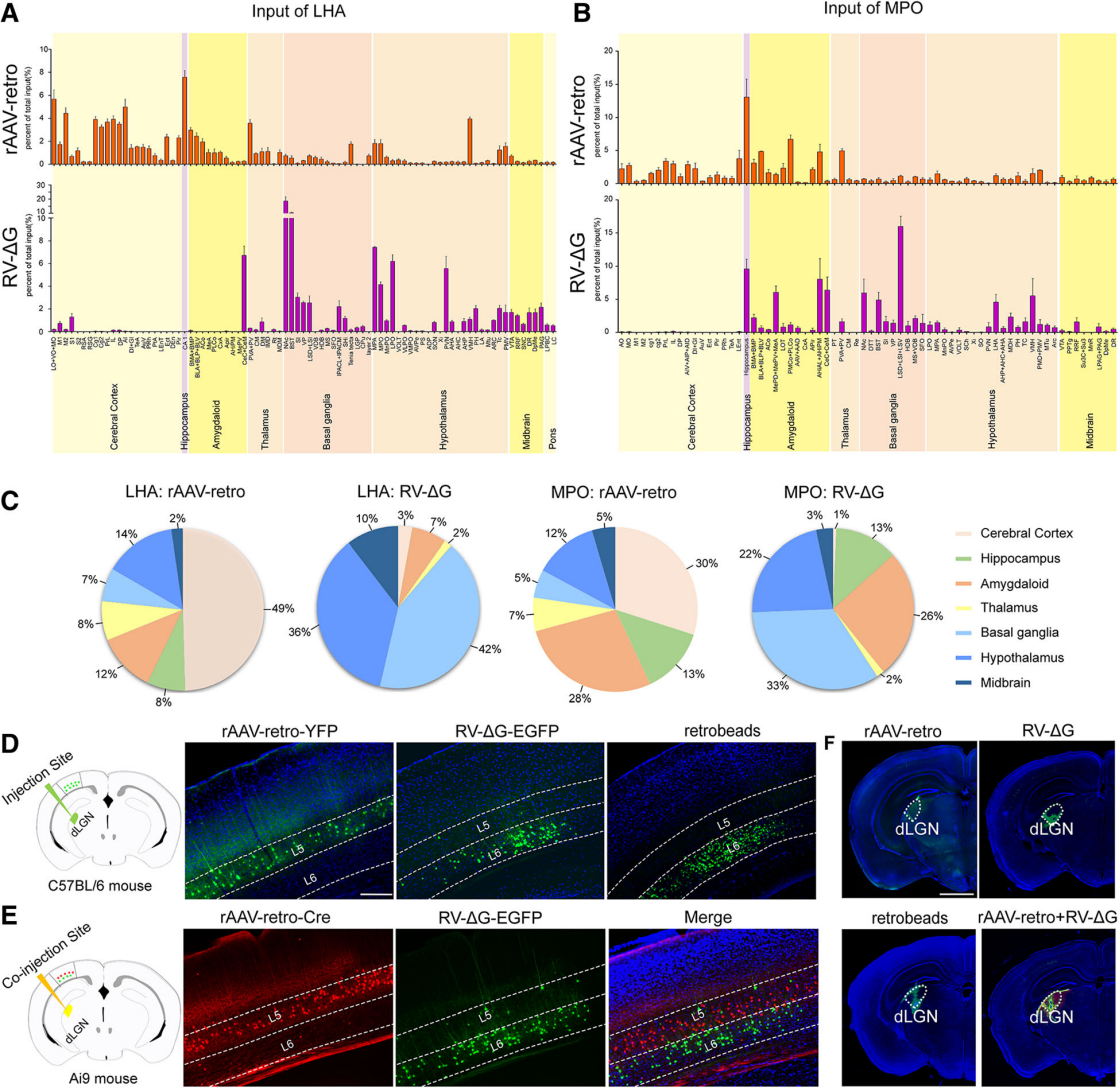
Labeling preference of rAAV2-retro and RV-∆G viral tracers in certain brain region and cortical layer. (**a-b**) Whole-brain quantification analysis of the retrograde labeling of input circuits to the LHA (A) and MPO (B) following injection of rAAV2-retro-Cre-tagBFP and RV-∆G-EGFP. The number of labeled neurons in each nucleus on the side ipsilateral to the injection site was quantified and normalized to the total number of ipsilateral labeled neurons for each mouse (n=3). (**c)** The percentage of labeled input brain regions to LHA and MPO was represented as pie chart to indicate the labeling preference of rAAV2-retro in cerebral cortex, and RV-∆G in basal ganglia and hypothalamus. (**d**) Retrograde tracers of rAAV2-retro-YFP, RV-∆G-EGFP, and green retrobeads were separately injected into the dLGN for retrograde labeling of upstream corticothalamic neurons in the primary visual cortex (V1). The corticothalamic neurons in V1 labeled by rAAV2-retro-YFP were predominately localized in the fifth layer (L5). In contrast, both RV-∆G-EGFP and green retrobeads mostly labeled corticothalamic neurons in the sixth layer (L6) of V1. (**E**) Retrograde tracers of rAAV2-retro-Cre-tagBFP and RV-∆G-EGFP were co-injected into the dLGN of Ai9 mice for simultaneously labeling of upstream corticothalamic neurons in V1. The rAAV2-retro-Cre-tagBFP predominately labeled V1 corticothalamic neurons in the fifth layer (L5), while RV-∆G-EGFP mostly labeled corticothalamic neurons in the sixth layer (L6) of V1. (**F**) The injection sites of rAAV2-retro-YFP, RV-∆G-EGFP, green retrobeads, and co-injection of rAAV2-retro-Cre-tagBFP with RV-∆G-EGFP. Scale bars = 200 μm in D-E, 1 mm in F

To analyze the tracing preference of different retrograde viral tracers based on above results, we summarized all the inputs of LHA and MPO labeled by rAAV2-retro-Cre-tagBFP and RV-∆G-EGFP throughout the whole brain. Full names of abbreviation for brain structures are listed in Table 1. As shown in Fig. 5a-c, rAAV2-retro-Cre-tagBFP preferentially labeled the cerebral cortex, while RV-∆G-EGFP preferentially labeled basal ganglia and hypothalamus. During neural circuit study, such labeling preference will provide important guidance for proper use of rAAV2-retro and RV-∆G tracers in order to reach optimal labeling. Notably, we even observed a striking preference for specific cortical layer by rAAV2-retro and RV-∆G when they were (co)-injected into dorsal lateral geniculate nucleus (dLGN) (Fig. 5f) to label corticothalamic neurons of the primary visual cortex (V1). As shown in Figure 5d, positive labeling was predominately presented in the fifth layer (L5) of V1 when rAAV2-retro-YFP was used; while, the positive labeling in V1 was mostly located within the sixth layer (L6) when RV-∆G-EGFP and green retrobeads were used. Further co-injection of rAAV2-retro-Cre-tagBFP and RV-∆G-EGFP into dLGN confirmed the layer preference of these two viral tracers (Fig. 5e).

Considering the labeling discrepancy among different retrograde viral tracers, the retrograde tracing using rAAV2-retro or RV-∆G alone can only reveal a portion of the direct upstream regions. Thus, it would be necessary to combine the tracing data of both tracers to obtain more comprehensive input mapping of a certain nucleus. In this regard, we collected all the tracing data from rAAV2-retro and RV-∆G and delineated a more comprehensive map of LHA direct upstream circuits, which provides an important reference for exploring the function of LHA circuit, such as feeding, arousal, pain perception, body temperature regulation, digestive function (Additional file 2: Figure S2).

### Retrograde Labeling using RV-∆G in RV-resistant Regions via the EnvA-TVA Pseudotype System

**Fig. 6:**
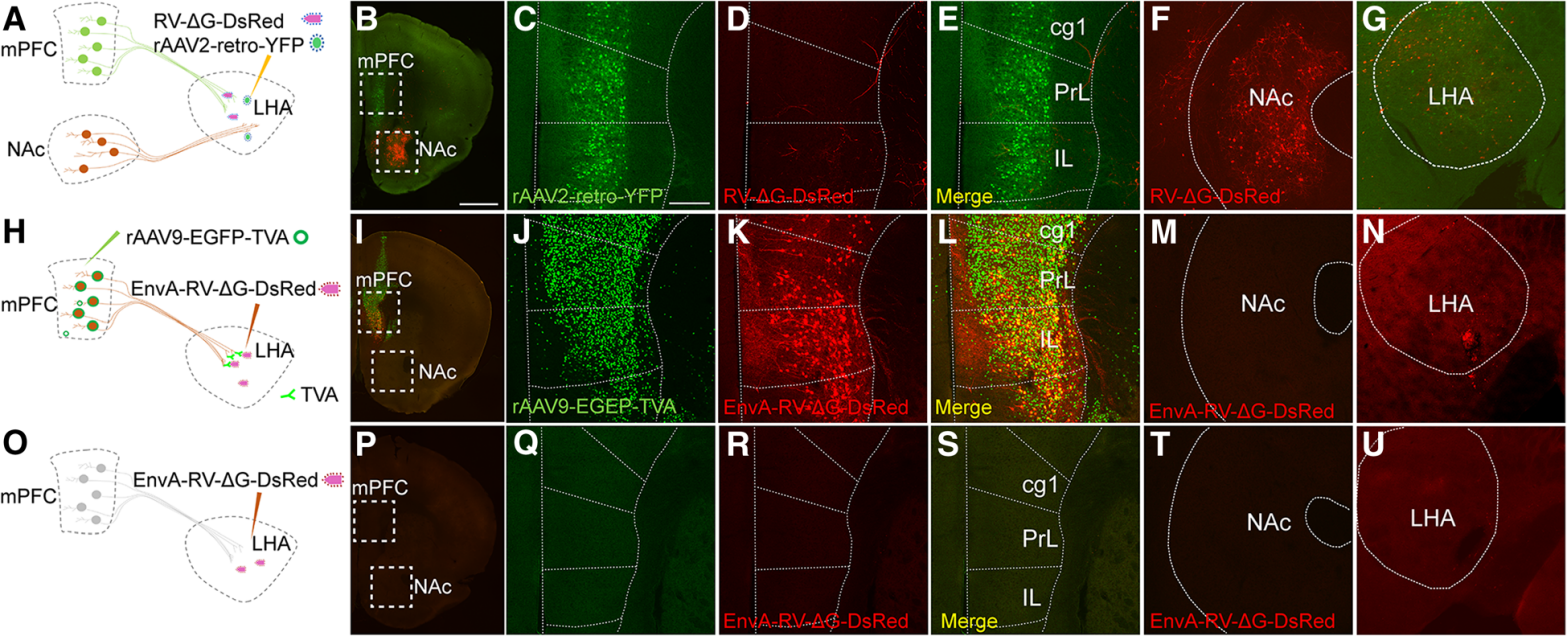
Complementing TVA receptor expression in the mPFC neurons enables EnvA pseudotyped RV infection. (**a**) Schematic diagram depicting the co-injection of rAAV2-retro-YFP and RV-∆G-DsRed into the LHA. (**b-f**) Co-injection of rAAV2-retro-YFP and RV-∆G-DsRed into the LHA labeled different input nuclei. The mPFC was labeled by rAAV2-retro-YFP and NAc was labeled by RV-∆G-DsRed. (**g**) The injection site for rAAV2-retro-YFP and RV-∆G-DsRed in LHA. (**h**) Schematic diagram depicting the injection of helper AAV expressing TVA (rAAV9-EGFP-TVA) to enable EnvA pseudotyped RV (EnvA-RV-∆G-DsRed) infection in the mPFC. (**i-m**) Complementation of TVA receptor expression in mPFC neurons enabled EnvA-RV-∆G-DsRed infection in the mPFC. No positive labeling of EnvA-RV-∆G-DsRed was detected in NAc. (**n**) The injection site for EnvA-RV-∆G-DsRed in LHA. (**o**) Schematic diagram depicting the injection of EnvA-RV-∆G-DsRed into the LHA without helper rAAV9-EGFP-TVA. (**p-t**) No signals were observed in the mPFC following injection of EnvA-RV-∆G-DsRed alone. (**u**) The injection site for EnvA-RV-∆G-DsRed in LHA. Scale bars =1 mm for B, I, P; 200 μm for magnification images

The aforementioned results indicated that different retrograde viruses are associated with differential circuit labeling, which are most likely due to the differential expression of specific receptors for viral invasion. The rare infection of RV-∆G-EGFP in the region of the mPFC (Figs. 3, 4, 5, Additional file 1: Figure S1) upstream from the LHA might be due to the absence of RV receptors in the mPFC neurons of this circuit. To examine this hypothesis, we employed avian sarcoma leukosis virus receptor TVA expressed by adeno-associated virus (rAAV9-EGFP-TVA) and avian sarcoma leukosis virus envelope protein (EnvA) pseudotyped RV-∆G (EnvA-RV-∆G-DsRed) to enable RV-∆G labeling of the mPFC from LHA (Fig. 6). To this end, rAAV9-EGFP-TVA was firstly injected into the mPFC to over-express TVA in mPFC neurons and their terminals. Then, EnvA-RV-∆G-DsRed was injected into the LHA (Fig. 6h). As compared with rare labeling of RV-∆G-DsRed in mPFC from LHA (Fig. 6a-g), expression of TVA in mPFC neuron terminals at the LHA could indeed lead to the successful labeling of EnvA-RV-∆G-DsRed to these mPFC RV-resistant neurons (Fig. 6h-n). In accordance with this finding, we observed that a large number of DsRed-positive neurons in the mPFC were overlapped with rAAV9-EGFP-TVA infected neurons (Fig. 6i-l). As a control, we performed the same experiment without injecting TVA into the mPFC, following which no neurons from the LHA were labeled by EnvA-RV-∆G-DsRed (Fig. 6o-u). Similarly, the L5 corticothalamic neurons in V1 which was resistant to RV-∆G, can also be successfully labeled by EnvA-RV-∆G-DsRed from dLGN through complementation of TVA receptor into V1 cortex (Additional file 3: Figure S3). Taken together, these data demonstrate that the exogenous expression of TVA receptor can indeed enable EnvA-pseudotyped RV to successfully enter the axons of RV-resistant neurons, supporting the notion that the resistance of these neurons to RV-∆G is due to the absence of RV receptors.

### Screening of Candidate Genes Contributing to Neurotropism via Single-cell Transcriptome Sequencing

**Fig. 7:**
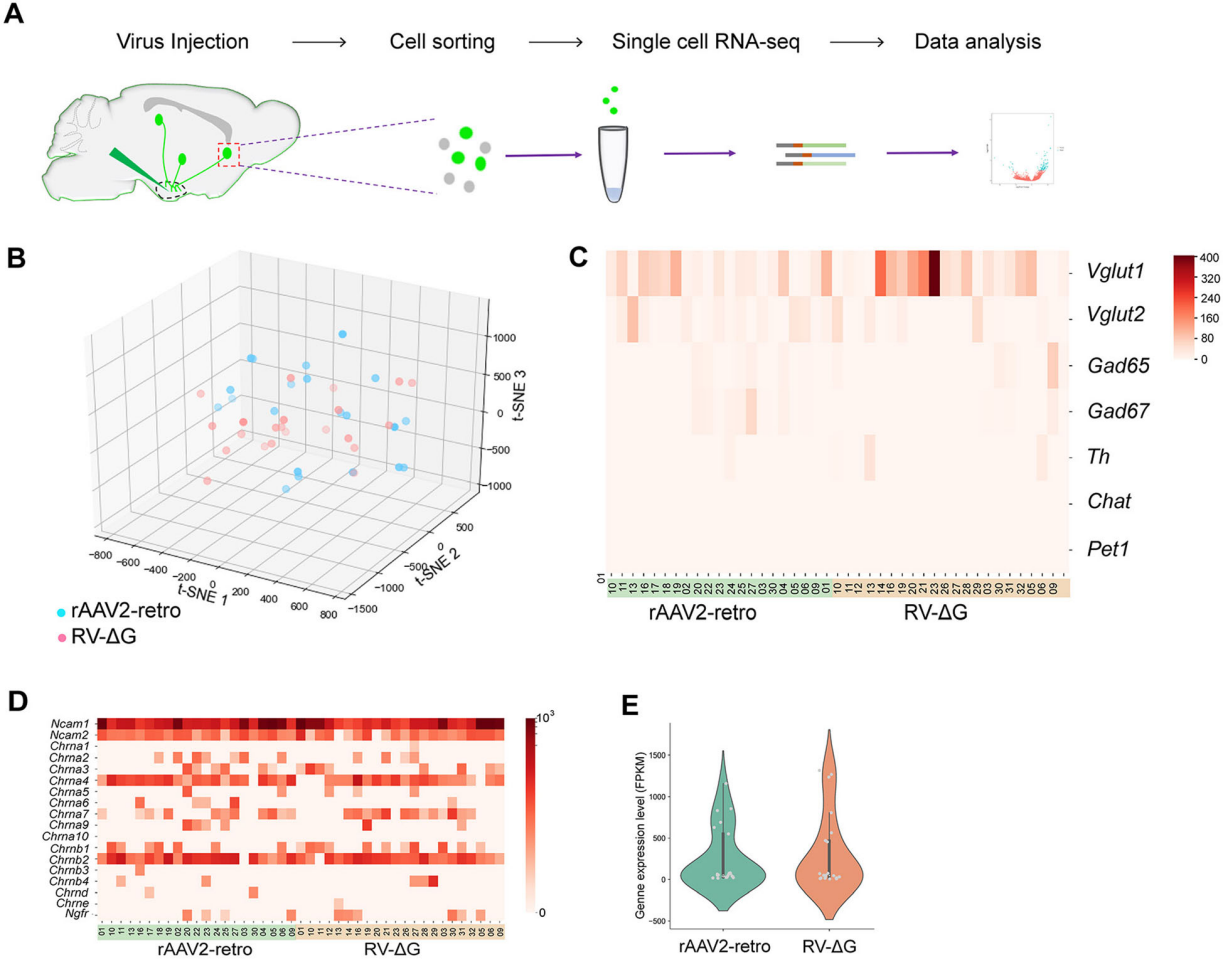
Gene profiling analysis of rAAV2-retro-labeled and RV-∆G-labeled neurons via single-cell RNA sequencing. (**a**) Flow chart of single-cell isolation and RNA sequencing. (**b**) Gene cluster analysis of the neuronal groups infected by rAAV2-retro-Cre-tagBFP and RV-∆G-EGFP. (**c**) Expression of neuronal marker genes including *VLGUT1*, *VGLUT2*, *GAD65*, *GAD67*, *TH*, *ChAT*, and *Pet1* in the rAAV2-retro-Cre-tagBFP and RV-∆G-EGFP groups. (**d**) Expression heat-map of potential receptor candidates for rabies virus, including *Ncam*, *Chrna*, and *Ngfr*, in each sample from rAAV2-retro and RV-∆G labeled groups. (**e**) These are no significant difference of *Ncam1* expression between rAAV2-retro and RV-∆G labeled groups, P=0.088. *Chrna*, nicotinic acetylcholine receptor gene; *Ncam*, neural cell adhesion molecule gene; Ngfr: Nerve growth factor receptor

To investigate the heterogeneous gene expression profiles of the neurons labeled by different retrograde viral tracers, which may have contributed to the observed differences in viral neurotropism, we performed single-cell transcriptome sequencing of the infected neurons (Fig. 7a). First, rAAV2-retro-Cre-tagBFP and RV-∆G-EGFP were parallelly injected into different nuclei of the hypothalamus, thalamus, hippocampus, and amygdala in Ai9 reporter mice, respectively. The retrogradely labeled neurons were then subjected to single neuron isolation, following which 29 groups of rAAV2-retro-Cre-tagBFP-labeled neurons and 30 groups of RV-∆G-EGFP-labeled neurons were collected. To ensure the reliability of the sequencing data, we filtered the samples using a quantity threshold of 25% and further analyzed 21 groups of rAAV2-retro-Cre-tagBFP-labeled neurons and 22 groups of RV-∆G-EGFP-labeled neurons with high quality, of which more than 10,000 gene transcripts were captured in each single RNA-seq data (Additional file 4: Figure S4A).

After gene sequencing and mapping, we performed t-SNE dimension reduction analysis on the single-cell RNA-seq expression data to cluster the rAAV2-retro-Cre-tagBFP- and RV-∆G-EGFP-infected neurons based on their gene expression profiles. As shown in Fig. 7b, the colored dots representing different groups of rAAV2-retro-Cre-tagBFP- and RV-∆G-EGFP-infected neurons were allocated according to their gene profiles, which demonstrated that there was no obvious pattern of distribution between the rAAV2-retro-Cre-tagBFP and RV-∆G-EGFP-labeled neuron groups. Next, we analyzed the expression of the specific marker genes for different types of neurons. No significant difference of these genes expression was observed between rAAV2-retro-Cre-tagBFP and RV-∆G-EGFP groups (VLGUT1: P=0.5134, VGLUT2: P=0.5327, GAD65: P=0.1138, GAD67: P=0.1217, TH: P=0.2231, ChAT: P=0.3266, and Pet1: P=0.3579), which indicated that both rAAV2-retro-Cre-tagBFP and RV-∆G-EGFP can infect all types of neurons (Fig. 7c).

Several potential receptor candidates for rabies virus have been reported, including nicotinic acetylcholine receptors (nAChR), neural cell adhesion molecules (NCAMs), and p75NTR/Ngfr [69, 70]. We then analyzed the expression of RV receptor candidate genes in all the groups of RV-∆G-EGFP- and rAAV2-retro-Cre-tagBFP-labeled neurons (Fig. 7d). Our data indicated that only the *Ncam1* gene was expressed in all groups of RV-infected neurons (Fig. 7d). However, no significant differences in *Ncam1* expression were observed between RV-∆G-EGFP- and rAAV2-retro-Cre-tagBFP-labeled neurons (Fig. 7e). Meanwhile, we did not observed exclusive expression of *Ncam*, *nAChR*, and *Ngfr* gene in RV-∆G infected neuron groups (Fig. 7d), implying these receptor candidates may not be the vital factors for RV-∆G neurotropism.

Since the receptors that mediate virus invasion should theoretically be synapse-specific proteins present on the cell membrane, the potential viral receptor candidate genes should most likely meet these three criteria: (1) be expressed in all groups of neurons labeled by a specific virus; (2) be membrane-specific; and (3) be synapse-specific. Thus, we developed a bioinformatics analysis pipeline to screen the potential viral receptors as shown in Additional file 4: Figure S4B. Finally, we identified 682 and 527 synapse-specific membrane protein genes expressed in all rAAV2-retro-Cre-tagBFP and RV-∆G-EGFP groups (Additional file 4: Figure S4C), respectively. Gene ontology (GO) analysis revealed that the receptor candidate genes are enriched in several pathways, including the "cell projection", "synaptic cleft", "cell-cell junction", and "postsynapse" pathways (Additional file 4: Figure S4D-S4E).

### Differences in the Neurotoxicity of rAAV2-retro and RV-∆G Tracers

**Fig. 8:**
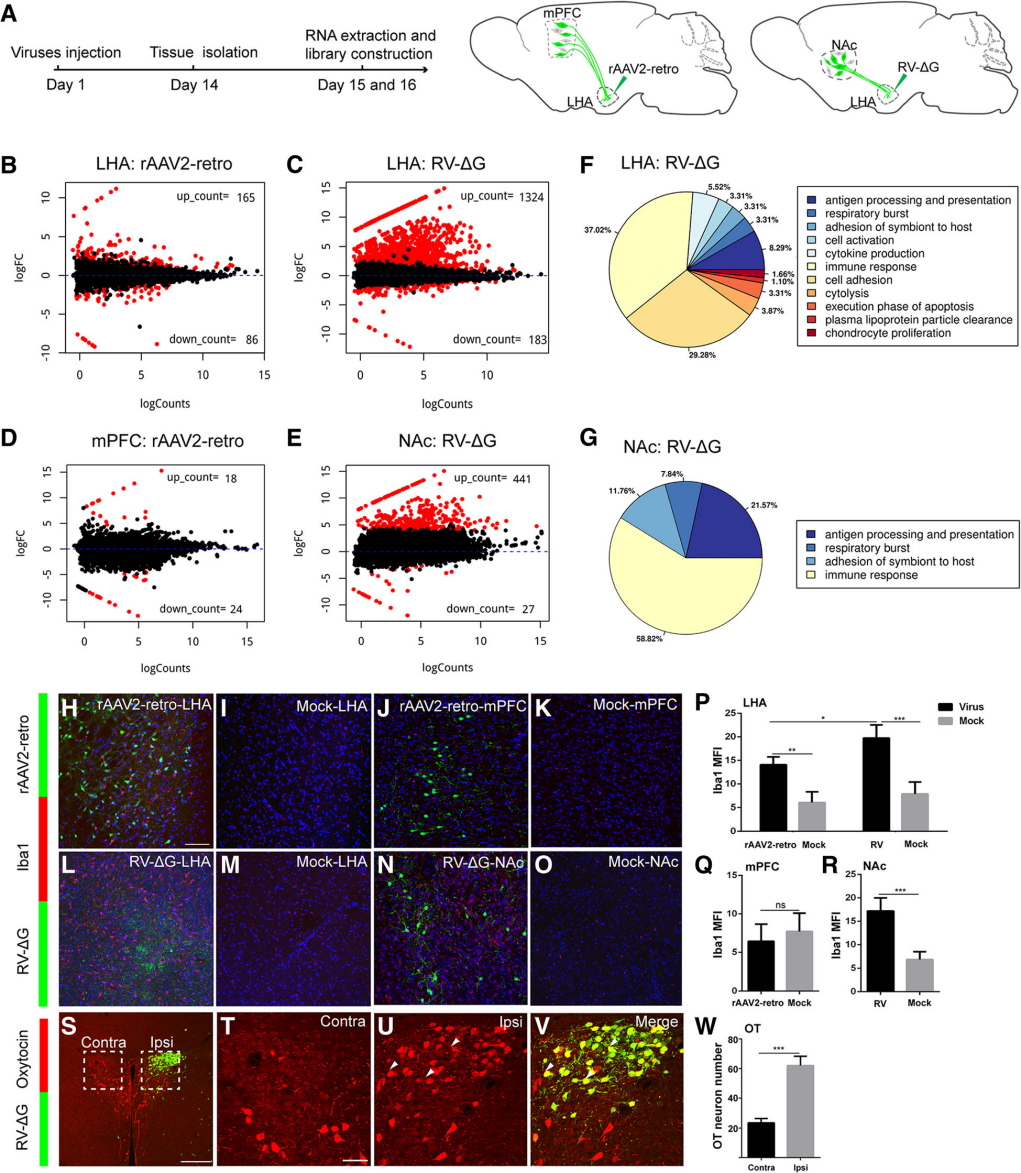
Analysis of neurotoxicity induced by rAAV2-retro and RV-ΔG at injection sites and retrogradely labeled nuclei. (**a**) Schematic diagram of virus injection and tissue extraction for RNA sequencing. (**b**) Injection of rAAV2-retro-YFP into the LHA caused alterations in gene expression profiles. (**c**) Injection of RV-∆G-EGFP into the LHA caused alterations in gene expression profiles. (**d**) Gene profile alterations in retrogradely labeled mPFC following injection of rAAV2-retro-YFP into the LHA. Red dots represent the genes with significant change, while the black dots represent the genes without significant change. (**e**) Gene profile alterations in retrogradely labeled NAc following injection of RV-∆G-EGFP into the LHA. (**f-g**) GO-term pathway analysis of differentially expressed genes at the site of RV-∆G-EGFP injection (LHA) and in retrogradely labeled nucleus (NAc). (**h-i**) Immunostaining for the microglial marker Iba1 in the rAAV2-retro-YFP group. Iba1-positive cells were observed on the injection site, but barely in the PBS injected (Mock) LHA. (**j-k**) No activated microglia was detected in the ipsilateral mPFC following injection of rAAV2-retro-YFP or PBS (Mock) into the LHA. (**l-m**) Intense microglial activation was observed at the injection site when RV-∆G-EGFP was injected into the LHA, while few signals were observed in the PBS injected (Mock) LHA. (**n-o)** Intense microglial activation was observed in the retrogradely labeled ipsilateral NAc following injection of RV-∆G-EGFP into the LHA, but not in the ipsilateral NAc of Mock control. (**p**) Quantification of mean fluorescence intensity (Mean±SEM) of Iba1 in LHA after virus injection. rAAV2-retro-YFP: 14.09±0.8373, Mock: 6.059±1.132, rAAV2-retro-YFP vs Mock: P=0.0013; RV-∆G-EGFP: 19.75±1.403, Mock: 7.885±1.265, RV-∆G-EGFP vs Mock: P = 0.0008; rAAV2-retro-YFP vs RV-∆G-EGFP: P = 0.0133; *n* = 4, n is mice number. (**q**) Quantification of mean fluorescence intensity (Mean±SEM) of Iba1 in mPFC injected with rAAV2-retro-YFP and PBS. rAAV2-retro-YFP: 6.438±1.114, PBS: 7.713±1.192, P = 0.4639, *n* = 4. (**r**) Quantification of mean fluorescence intensity (Mean±SEM) of Iba1 in NAc injected with RV-∆G-EGFP and PBS. RV-∆G-EGFP: 17.20±1.408, Mock: 6.883±0.8132, P = 0.0007; *n* = 4. (**s**) Immunostaining for oxytocin in PVN neurons 7 days following the injection of RV-∆G-EGFP into the LHA. (**t, u**) Boxed areas in S were magnified to show oxytocin-positive neurons in the ipsilateral and contralateral PVN to the virus injection side. (**v**) Higher-magnification images of the dashed box depicting the ipsilateral PVN in S with green color and merged color. Green, GFP expressed by RV-∆G-EGFP; red, oxytocin immunostaining signal. (**w**) Quantification of oxytocin-positive neurons (Mean±SEM) in the ipsilateral and contralateral PVN. Contra: 23.50±1.443, Ipsi: 62.00±3.136, P=0.0001, *n* = 4. MFI, mean fluorescence intensity; Ipsi, ipsilateral; Contra, contralateral. n is mice number. Scale bars =200 μm for S; 100 μm for H-O; 50 μm for T-V. Unpaired t-tests, *P < 0.05, **P < 0.01, ***P < 0.001

As neurotropic viruses have also been extensively applied in the recording of circuit functions, as well as in circuital manipulation and gene profiling studies, we aimed to systematically analyze the neurotoxicity of these viruses at the injection site and in retrogradely labeled nuclei. First, the injection site (LHA), rAAV2-retro-YFP-labeled nuclei (mPFC), and RV-∆G-EGFP-labeled nuclei (NAc) were dissected from the experimental and control groups 2 weeks after injection (Fig. 8a). RNA was then extracted from the tissues of different nuclei and subjected to library construction and RNA sequencing. Bioinformatic analysis of the gene profiles from different groups revealed that infection with retrograde viral tracers can indeed lead to alterations in gene expression, as shown in the volcano plots in Fig. 8b-e. RV-∆G-EGFP infection caused greater changes in gene expression both at the injection site and in retrogradely labeled nuclei than rAAV2-retro-YFP infection, indicating that the impact of RV-∆G-EGFP infection on gene expression is more pronounced (Fig. 8b-e). We also performed Gene ontology (GO) analysis for the differentially expressed genes in all groups, which revealed that RV-∆G-EGFP infection mainly influenced genes associated with the immune response, cell adhesion, and antigen processing/presentation both at the injection site and in retrogradely labeled nuclei (Fig. 8f-g). In contrast, no genes associated with specific terms were enriched at the injection site or in retrogradely labeled nuclei following rAAV2-retro injection. Therefore, we then performed KEGG (Kyoto Encyclopedia of Genes and Genomes) pathway analysis of the differentially expressed genes in all groups, of which the data were shown in Additional file 5: Figure S5.

As viral infection in the CNS can activate microglial cells [65], we performed Iba1 immunohistochemistry to evaluate microglial activation induced by RV-∆G and rAAV2-retro infection at the injection site and in retrogradely labeled nuclei. As shown in Figure 8l-o, p, r, Additional file 6: S6C-S6D, microglial infiltration was markedly increased at the injection site (LHA) and in the retrogradely traced region (NAc) following RV-∆G injection. While microglial activation due to rAAV2-retro infection was significantly milder (Fig. 8h-k, p, q, Additional file 6: Figure S6A-S6B) even after 30 days, indicating that RV-∆G is much more toxic to both the injection site and retrogradely labeled regions. In contrast, no significant change of astrocyte marker GFAP (glial fibrillary acidic protein) was observed in RV-∆G and rAAV2-retro infection both at the injection sites and in retrogradely labeled nuclei (Additional file 6: Figure S6E-S6O). We further investigated the effect of RV infection in neurons of the PVN, a central nucleus that secretes various neuroendocrine hormones, via an immunostaining analysis of gene expression for different hormones. We thus examined oxytocin and vasopressin expression in the PVN 7 days and 14 days after RV-∆G-EGFP injection in the LHA, respectively. Our findings indicated that more than 90% of RV-labeled PVN neurons expressed oxytocin, and that the number of oxytocin-positive neurons was significantly increased in the area of the PVN ipsilateral to the site of RV injection, relative to the number observed on the contralateral side (Fig. 8s-w, Additional file 6: Figure S6P-S6T). These findings suggest that RV infection can lead to ectopic oxytocin expression in PVN neurons. In contrast, the expression of vasopressin was not altered by RV-∆G-EGFP infection (Additional file 6: Figure S6U-S6Y).

### Proof of Concept for Target-specific, Higher-order Circuit Tracing by Combining Different Viral Tracers

**Fig. 9:**
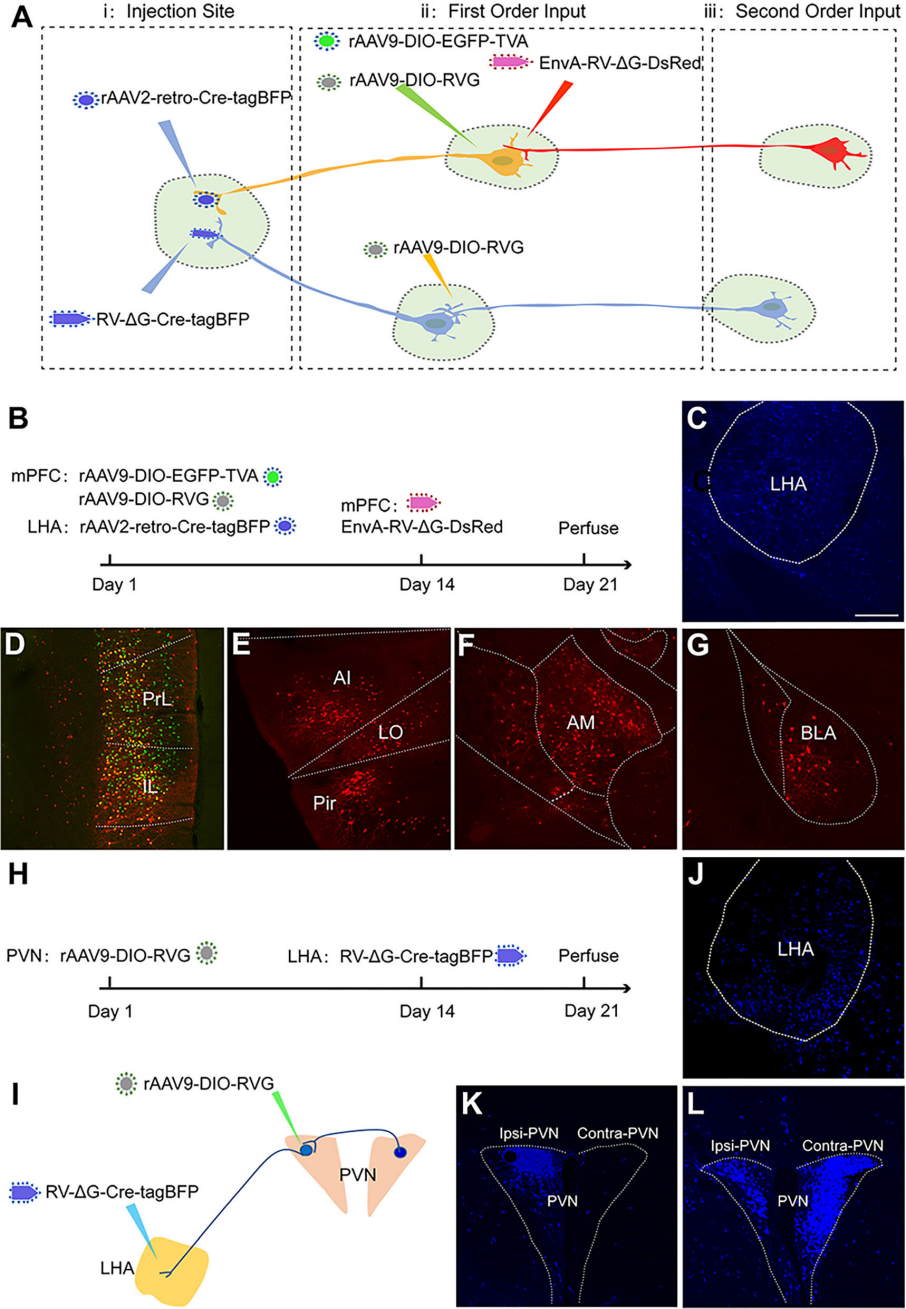
Higher-order circuit tracing using a combination of different tracing viruses. (**a**) Strategy for mapping the higher-order input circuits of target nuclei by combining different viral tracers. (**b**) Diagram of higher-order circuit mapping of the LHA by combining rAAV2-retro-Cre-tagBFP, rAAV9-DIO-EGFP-TVA, rAAV9-DIO-RVG, and EnvA pseudotyped RV-∆G-DsRed. (**c**) Injected site for rAAV2-retro-Cre-tagBFP in LHA. (**d**) Direct upstream neurons from the LHA (first order) were labeled with both green and red fluorescence in the PrL and IL of the mPFC (also called starter neurons). Green, TVA-positive neuron; red, EnvA-RV-∆G-DsRed-positive neuron; yellow, starter neurons. (**e-g**) Nuclei upstream from the starter neurons (second order) were labeled with only red fluorescence in the AI, LO, Pir (E), AM (F), and BLA (G). (**h-i**) Diagram of the higher-order circuit map of the LHA obtained by combining rAAV9-DIO-RVG and RV-∆G-Cre-tagBFP labeling in the PVN. (**j**) Injected site for RV-∆G-Cre-tagBFP. (**k**) RV-∆G-Cre-tagBFP retrogradely labeled only neurons in the ipsilateral PVN. (**l**) Complementing RV-G by injecting AAV-DIO-RVG into the ipsilateral PVN resulted in the labeling of second-order neurons in the contralateral PVN. Scale bars = 200 μm.

Our data demonstrated that application of rAAV2-retro or RV-∆G for retrograde tracing only reveals a portion of the direct upstream regions. Thus, to achieve a more comprehensive map of higher-order circuits involving a specific target nucleus, we proposed a strategy by combing the rAAV2-retro, rAAV, and rRV tracers as shown in Fig. 9a. In this strategy, rAAV2-retro-Cre-tagBFP and RV-∆G-Cre-tagBFP are firstly injected into the target nuclei (i), respectively, to trace and deliver Cre to the direct upstream connected nucleus (ii). Cre-dependent rAAV9-DIO-EGFP-TVA and rAAV9-DIO-RVG, and EnvA-RV-∆G-DsRed are injected into rAAV2-retro-Cre-tagBFP labeled nucleus ii for mono-trans-synaptic labeling of the secondary input circuits. In the upstream nucleus ii labeled by RV-∆G-Cre-tagBFP, rAAV9-DIO-RVG is injected into nucleus ii to ensure that the RV could trans-synaptically hop to the second-order circuits of target i. Accordingly, we injected rAAV2-retro-Cre-tagBFP into the LHA and the combination of rAAV9-DIO-EGFP-TVA and rAAV9-DIO-RVG into the mPFC (Fig. 9b). 14 days later, EnvA-RV-∆G-DsRed was injected into the mPFC to trace the secondary upstream circuits (Fig. 9b). As shown in Figure 9c-d, rAAV2-retro-Cre-tagBFP specifically labeled mPFC neurons targeting the LHA, and triggered expression of TVA and RVG in these neurons, which mediated the infection of EnvA-RV-∆G-DsRed and further initiated mono-trans-synaptic tracing of the secondary upstream circuits of the LHA. DsRed-positive neurons infected by EnvA-RV-∆G-DsRed were observed in the local mPFC, agranular insular cortex (AI), lateral orbital cortex (LO), piriform cortex (Pir), anteromedial thalamic nucleus (AM), and basolateral amygdaloid nucleus (BLA), which provide second-order input to the LHA (Fig. 9c-g).

Moreover, to map higher-order upstream circuits of the LHA traced by RV-∆G, we injected rAAV9-DIO-RVG into the PVN 14 days prior to the injection of RV-∆G-Cre-tagBFP into the LHA (Fig. 9h-j). As shown in Figure 9k-l, PVN neurons targeting the LHA were retrogradely labeled by RV-∆G-Cre-tagBFP, leading to the expression of RV-G in these neurons and initiating mono-trans-synaptic tracing of upstream nuclei within the PVN-LHA circuit 7 days after RV-∆G-Cre-tagBFP injection (e.g., contralateral PVN). Taken together, these data provide a proof-of-concept for more comprehensive higher-order circuit mapping of a specific target nucleus via a combination of rAAV2-retro, rAAV and rRV.

## Discussion

Viruses have evolved to invade the central nervous system (CNS) via different strategies. As various potential receptors for different neurotropic viruses have been discovered, different viruses may exhibit distinct patterns of tropism in the nervous system. In this line, our findings demonstrated that the tropism of retrograde multi-trans-synaptic tracers such as pseudorabies virus (PRV) and rabies virus (RV) within the CNS are indeed differential. Thus, the mono-synaptic tracers modified from these neurotropic viruses should in principle exhibit preferential labeling of specific circuits. In this study, we engineered a TK-deleted PRV expressing GFP (PRV-∆TK-∆US9-EGFP) and a glycoprotein-deleted (ΔG) RV, and compared their neurotropic actions in the rodent brain. However, the fluorescence signal of PRV-∆TK-∆US9-EGFP was hardly detectable, most likely due to impairments in viral replication following TK deletion. Therefore, we focused on the most widely used retrograde mono-synaptic tracers: glycoprotein-deleted (∆G) RV, rAAV2-retro, and retrobeads. Our data revealed that these tracers indeed exhibited preferential retrograde circuit labeling. The rAAV2-retro-labeled neurons projecting to the lateral hypothalamic area (LHA) and medial preoptic nucleus (MPO) were preferentially localized in the cerebral cortex. In contrast, those labeled by RV-∆G were primarily localized in the basal ganglia and hypothalamus.

Notably, we demonstrated rAAV2-retro predominately labeled the layer 5 (L5) of primary visual cortex (V1) from dorsal lateral geniculate nucleus (dLGN), whereas RV-∆G and green retrobeads mostly labeled the L6 of V1. As this preference was also observed when these two viruses were co-injected, the distinct labeling pattern is not due to the injection variation of different virus. The labeling of V1 layer 6 corticothalamic neurons by RV-ΔG and retro-bead is consistent with previous findings in which similar RV-ΔG or retro-beads tracer was injected into dLGN and specifically labeled layer 6 corticothalamic neurons of V1 [44, 71]. Few studies report the labeling of layer 5 corticothalamic neurons in V1 from dLGN, possibly due to the limit of current circuit tracers, while it has been demonstrated that layer 5 neurons of V1 cortex can project to dLGN [72]. Meanwhile, TVA supplementation in V1 helped EnvA pseudotyped RV-ΔG injected in the dLGN to trace back layer 5 corticothalamic neurons, further confirming the projection of V1 layer 5 neurons to dLGN, and also supporting the notion that resistance of neurons to RV-∆G is due to the absence of RV receptors.

During neurotropic comparison between monosynaptic viral tracers, the rAAV2-retro virus with different promoters, including rAAV2-retro-YFP (ef1α promoter) and rAAV2-retro-Cre-tagBFP (CMV promoter), were used. Although the two rAAV2-retro viruses gave the mostly consistent results, there is still mild difference such as labeling in PVN, Xi nucleus, and NAc by rAAV2-retro-CMV-Cre-tagBFP but not rAAV2-retro-YFP. While different promoters (CMV or ef1α) could influence gene expression in certain level, the most possible reason for this labeling difference might be due to the amplification effect caused by Cre. Nevertheless, the different promoters did not affect the neurotropism property of rAAV2-retro tracer and its labeling discrepancy in comparison with RV-∆G tracer.

The distinct tropism of these tracers in the CNS may have been due to differences in the presence of the corresponding receptors that initiate their entrance into neurons. It has been reported that canine adenovirus 2 (CAV2) infects cells by binding to coxsackievirus and adenovirus receptor (CAR) [22]. The complementation of CAR receptor can increase the CAV2 retrogradly labelling efficiency [67]. Meanwhile our data show that the exogenous expression of TVA receptor can indeed enable EnvA-pseudotyped RV to successfully enter the axons of RV-resistant neurons. While several genes including the *nAChR*, *NCAM*, and the *p75NTR/Ngfr* has been considered as potential RV receptors [69, 70], we did not observe significant difference of the expression level of these genes between rAAV2-retro and RV-∆G groups. These potential receptors for RV infection may be not the definitive factor for RV neurotropism in central nervous system. In accordance with the notion that differences in tropism may be due to differences in the expression of viral receptors, we showed that exogenous expression of the TVA receptor in RV-∆G-resistant nucleus can indeed enable the EnvA-pseudotyped RV infection in this nucleus. To investigate the molecular machinery underlying viral tropism, we thus analyzed the gene expression profiles of 22 groups of RV-∆G-infected neurons and 21 groups of rAAV2-retro-infected neurons with high quality of single-cell RNA sequence data. In theory, potential viral receptors should be synaptic membrane proteins that are expressed in all labeled neurons. Therefore, we screened RV and rAAV2-retro candidate receptor genes using different databases and identified 527 genes as potential receptors for RV invasion and 682 genes as potential receptors for rAAV2-retro infection. Previous studies have reported that factors other than viral receptors may also influence the tropism of different viruses. Albisetti et al. demonstrated that certain receptors mediate virus entry into sensory neurons of the dorsal root ganglia (DRG), and that host-limiting factors may repress viral transcription or replication, thus influencing the ultimate efficiency of circuit mapping [65]. Thus, future studies should employ other biochemical and virological experiments to identify the exact receptors for these viruses or host-limiting factors, which may help to elucidate the mechanism of viral invasion and improve virus-based retrograde labeling techniques.

In addition to their role in neural circuit mapping, neurotropic viruses have been extensively utilized in the functional analysis and manipulation of specific neural circuits, in combination with Ca^2+^ indicators and optogenetic/chemogenetic techniques [52, 68]. Moreover, viral tracers expressing fluorescent proteins, tagged ribosomal subunits, or other functional proteins have enabled the isolation of target neurons and single-cell sequencing analysis of the transcriptome, translatome, and epigenome within a specific circuit [73, 74]. However, different viruses are associated with different levels of neurotoxicity in the host cells at different stages of infection. Thus, further studies are required to more fully reduce the toxicity of different viruses to avoid artifacts during neuronal manipulation, recording, and gene sequencing. Previous studies have indicated that neuronal characteristics remain largely unchanged 5-11 days after RV-∆G injection and can thus be applied for neuronal activity recording and optogenetics manipulation during this window [68]. However, neurons begin to die 16 days after RV-∆G injection [32]. In the present study, we investigated neurotoxicity at the injection site and in retrogradely labeled neurons using a more sensitive method: gene profiling analysis. Our data indicated that both RV-∆G and rAAV2-retro exert some influence at both the injection site and retrogradely labeled sites, although these changes were more profound for RV-∆G infection. Thus, such influences should be considered during functional recording, manipulation, and gene profiling analysis.

In our study, parallel comparison of neurotropism and neurotoxicity among RV-∆G and rAAV2-retro are performed at 14 days after injection, when viral labeled signals can reach high enough level for detection. Based on previous publication [32] and our experience, neural circuit labeling by RV-∆G will reach optimized results about 14 days after injection, when labeling efficiency are high and toxicity is relatively low. For rAAV and rAAV-retro, efficient labeling results can be reached around 2-3 weeks after injection [29]. Thus, we compared the toxicity within the time window of two weeks post virus injection to provide some reference for the toxicity caused by neural circuit tracing using different circuit tracers with comparable tracing effect. For PRV-∆TK-∆US9-EGFP, we speculate that undetectable expression of EGFP is mainly attributed to the low copy of PRV genome due to replication defect without TK gene, which is supported by a recent paper showing few GFP fluorescence in PRV-DIO-ΔTK-GFP infected input neurons without Cre recombinase [75].

Although rAAV2-retro is associated with lower levels of toxicity, it cannot replace RV-∆G for circuit tracing, as rAAV2-retro exhibit different patterns of tropism compared with RV-∆G and cannot be used for trans-synaptic circuit tracing. Several groups have successfully reduced RV cytotoxicity and increased the tropism by generating new RV tracers. The CVS-N2cΔG RV strain, the newly engineered self-inactivating ΔG-RV (SiR) and the recombinant RV without polymerase gene exhibits a significant reduction in neuronal toxicity [49–51]. The new deletion-mutant viral tracer CVS-N2cΔG derived from fixed rabies virus strain, is less toxic to neuron as compared with vaccine strain SAD-ΔG [51]. The engineering of a self-inactivating ΔG-RV (SiR) tracer via conditional modulation of viral nucleoprotein (N) stability [49], or abolishing the replication of RV viral genome by simultaneously deletion of glycoprotein (G) and RNA polymerase (L) gene [50], both dramatically decrease toxicity, which are better for neural circuits function manipulation. However, the rescue efficiency of these tracers is lower than that of vaccine strain SAD-ΔG, and preparation of these viral tracers requires a high level of expertise and is technically demanding, limiting their wide application in neural circuit study. Future studies are required to more fully elucidate the mechanisms underlying viral replication and toxicity, and then reduce the toxicity of the viral tracers by virus reverse genetic engineering such as precisely controlling the expression of cytotoxicity-related genes.

As the different viral tracers examined in the present study exhibited discrepant patterns of tropism, we reconstructed a more comprehensive map of LHA direct input circuits by combining the retrograde tracing data of both viral tracers. Combining this strategy with other technologies, such as optogenetics and electrophysiology, may greatly facilitate to more fully elucidate the functions of a nucleus. Moreover, we further provide proof-of-concept that the combination of these viruses can be used to delineate the higher-order inputs of a specific target nucleus. Using this strategy, researchers can create a more comprehensive map of direct upstream circuits, as well as the corresponding second-order circuits. Recent multidisciplinary work in the fields of virology, neuroscience, and biotechnology (e.g., protein engineering and development of new functional indicators) suggest that the number of available neural tracers with low toxicity and diverse functional applications will increase in the near future. Such advancements will not only improve our ability to systematically map, record activity within, and manipulate neural circuits, but may also enable us to elucidate the gene profiles and (epi) genetic architecture of circuits within the most sophisticated brain networks.

## Conclusions

In our study, we systematically evaluated the neurotropic discrepancy of different multi-trans-synaptic and mono-synaptic retrograde viral tracers including PRV, RV and rAAV2-retro. Mono-synaptic viral tracer rAAV2-retro displayed more preference in cerebral cortex, while RV-∆G showed more preference in basal ganglia and hypothalamus. Such distinct labeling preference indicates that RV-∆G can be first considered to map input from basal ganglia and hypothalamus, and rAAV2-retro be considered for input labeling from cerebral cortex. Combining data from RV-∆G and rAAV2-retro will provide a more comprehensive input map for precise neural circuit delineation of an interested brain nucleus. Remarkably, cortical layer preference was observed with rAAV2-retro in layer 5 and RV-∆G in layer 6 when they were applied to trace V1 corticothalamic neurons to dLGN. Layer specific labeling of V1 corticothalamic neurons by RV-∆G and rAAV2-retro will be utilized to dissect circuit map and function of corticothalamic neurons in specific layer. Both RV-∆G and rAAV2-retro exert neurotoxic influence at the injection sites and retrogradely labeled sites, with more profound effect of RV-∆G infection. It is necessary to take the tracing virus induced neurotoxicity into account especially for the functional study of neural circuit. Finally, a proof-of-concept strategy for more comprehensive high-order circuit tracing of a specific target nucleus was accomplished by combining rAAV2-retro, RV, and rAAV tracers. Our findings provide important reference for appropriate application of viral tracers in neural circuit research.

## Methods

### Ethics statement

The use of mice was approved by the Research Ethics Committee of Huazhong Agricultural University in Hubei, China (HZAUMO-2016-021). Preparation of viruses was performed in bio-safety level 2 (BSL-2) lab. All procedures were performed in accordance with the Guide for the Care and Use of Laboratory Animals of the Research Ethics Committee of Huazhong Agricultural University.

### Animals

Wild-type C57BL/6 mice and Ai9 reporter mice (B6.Cg-*Gt(ROSA)26Sor*^*tm9(CAG-tdTomato)Hze/J*^ (The Jackson Laboratory) [76]; provided by Prof. Yuqiang Ding of Tongji University, China) were housed under standard conditions, with food and water available *ad libitum*. Mice were maintained on a 12-h light/dark cycle at a temperature of 22–25°C. Mice aged between 8 and 10 weeks were used for virus injection.

### Viral construction and preparation

Viral vectors were constructed in accordance with the Biosafety Guidelines of the Huazhong Agricultural University Administrative Biosafety Committee on Laboratory. To generate mono-synaptic retrograde virus, the glycoprotein (G) of rabies virus was replaced with fluorescent protein to eliminate the ability of trans-synapse spreading. To construct recombinant glycoprotein (G)-deleted RV (rRV-∆G-EGFP, rRV-∆G-DsRed, rRV-∆G-Cre-tagBFP), enhanced green fluorescent protein (EGFP), DsRed, and Cre-T2A-tagBFP were subcloned into a pSAD-F3 backbone (provided by Dr. Fuqiang Xu of the Wuhan Institute of Physics and Mathematics, Chinese Academy of Science, and Dr. Edward Callaway of the SALK Institute, USA), respectively. The viruses were rescued as previously described [47]. Briefly, G-deleted RV plasmids and helper plasmids (expressing B19N, B19P, B19G, and B19L) were co-transfected into B7GG cells (provided by Dr. Xu and Dr. Callaway) and cultured for 7-10 days. The viral supernatant was then harvested and centrifuged at 70,000 *g* for 3 h, following which the samples were suspended in DMEM and spun on top of 20% sucrose solution. Finally, the recombinant RV was re-suspended in phosphate buffered saline (PBS), aliquoted, and stored at –80^o^C. The titer of recombinant RV was 4×10^9^ infectious particles per mL for RV-∆G-EGFP, 3×10^9^ infectious particles per mL for RV-∆G-DsRed, and 2×10^9^ infectious particles per mL for RV-∆G-Cre-tagBFP. EnvA-pseudotyped RV-∆G-DsRed was rescued in the BHK-EnvA cell line (provided by Dr. Xu and Dr. Callaway), at a titer of 3×10^9^ infectious particles per mL. CVS-B2C-EGFP with EGFP inserted between G- and L-coding sequences of RV-B2C strain (4x10^7^ infectious particles per mL) was prepared and provided by Prof. Ling Zhao (Huazhong Agricultural University, Wuhan, China).

Recombinant adeno-associated virus rAAV2-retro-YFP (2×10^12^ viral particles per mL), rAAV9-EGFP-TVA (6×10^12^ viral particles per mL), rAAV9-DIO-EGFP-TVA (6×10^12^ viral particles per mL), and rAAV9-DIO-RVG (6×10^12^ viral particles per mL) were packaged by BrainVTA (Wuhan, China). Plasmids for rAAV2-retro-Cre-tagBFP were generated from pAAV-CMV-GFP (Addgene number 67634) and rescued in 293T cells as previously described [77]. Because TK (thymidine kinase) gene is involved in viral replication and US9 is responsible for anterograde transport, a retrograde mono-synaptic tracing virus PRV-∆TK-∆US9-EGFP was obtained by deleting both TK and US9 genes, in which the location of US9 gene was replaced by CMV promoter and EGFP gene via homologous recombination on the BAC backbone of the Becker strain (provided by Dr. Lynn W. Enquist, Princeton University) as previously described [78]. PRV-152 (provided by Dr. Lynn W. Enquist), in which EGFP was driven by the CMV promoter in the Bartha strain [18], was amplified in PK-15 cells and concentrated at 70,000 *g* for 2 h at 4°C. The titers of PRV-∆TK-∆US9-EGFP and PRV-152 were 1×10^9^ infectious particles per mL and 5×10^9^ infectious particles per mL, respectively. All the viruses used were listed in Fig. 1.

### *In vivo* microinjection of viral and chemical tracers

Mice were anesthetized via an intraperitoneal injection of 65 mg/kg ketamine and 13 mg/kg xylazine, following which they were fastened into a stereotaxic apparatus (68030, RWD, China). A dental drill (78012, RWD, China) was used to create a small craniotomy opening in the exposed mouse skull. Viral and chemical tracers were injected at a rate of 10 nL/min by a glass micropipette with a tip diameter of 20–30 μm, which was connected to a micropump (53311, Stoelting, USA). The glass micropipette was held in place for 5 min prior to the injection and 10 min after the injection. For retrograde tracing of the lateral hypothalamic area (LHA) (anteroposterior (AP): –1.0 mm; mediolateral (ML): 1.22 mm; dorsoventral (DV): 4.93 mm), the dorsal part of the lateral geniculate nucleus (dLGN) (AP: –2.35 mm; ML: 2.26mm; DV: 2.70 mm), and medial preoptic nucleus (MPO) (AP: 0.15 mm; ML: 0.5 mm; DV: 4.7 mm), mice were injected with 50–200 nL of RV-∆G-EGFP, RV-∆G-Cre-tagBFP, rAAV2-retro-YFP, or rAAV2-retro-Cre-tagBFP and housed for 2 weeks to allow for fluorescence gene expression. Dye-based tracing was performed by injecting 50 nL of green-retrobeads (78G180, Lumafluor) into the LHA.

To help EnvA-pseudotyped RV-∆G-DsRed infection and visualization of mono-trans-synaptic tracing, 300 nL of rAAV9-EGFP-TVA virus was injected into the medial prefrontal cortex (mPFC) (AP: 1.6 mm; ML: 0.45 mm; DV: 0.8–1.2 mm) 21 days prior to injection of the EnvA-pseudotyped RV-∆G-DsRed virus (250 nL) into the LHA to guarantee the expression of the TVA receptor for EnvA-pseudotyped virus infection. EnvA-pseudotyped RV-∆G-DsRed virus was injected into the LHA of other wild-type (WT) mice as a control. Two weeks after the injection of EnvA-pseudotyped RV, mice were perfused for imaging analysis. PRV-152 and CVS-B2C-EGFP (2 μL each) were injected into the popliteal lymph nodes using a glass micropipette connected to a micro-syringe for trans-synaptic retrograde tracing. The glass micropipette was held in place for 5 min after the injection. Mice were perfused approximately 7-9 days after virus injection. All of the information on virus injection including titiers, injection volume, coordinates of injection sites are listed in Table 2. 

### Preparation, immunohistochemistry, and imaging of brain slices

Mice were anaesthetized and transcardially perfused with 4% paraformaldehyde (PFA) in PBS. Mouse brains were dissected and post-fixed in 4% PFA in PBS, following which they were embedded in 4% agarose in PBS and sectioned to a thickness of 50 μm using a semiautomatic vibratome (Leica VT1200, Germany). The slices were selected under fluorescence microscope according to Allen Mouse Brain Atlas. Brain slices were blocked with 3% BSA in PBS with 0.3% Triton X-100 for 2 h, following which they were incubated with primary antibody (anti-oxytocin: 1:1000, AB911, Millipore; anti-vasopressin: 1:1000, PC234L, Millipore; anti-Iba1: 1:500, ab178847, Abcam; anti-GFAP: 1:500, GB12096, Servicebio) overnight at 4°C. Slices were washed three times with PBS, following which they were incubated with the secondary antibody Alexa Fluor^TM^594 conjugated goat anti-rabbit immunoglobulin G (IgG) (1:1000, R37117, Invitrogen) for 1 h at 37°C. Finally, slices were counterstained with DAPI and imaged via confocal microscopy (FV1000, Olympus, Japan). To visualize neurons labeled with neurotropic viruses, brain slices were directly counterstained with DAPI and mounted on glass slides. All images were captured using an Olympus microscopy slide scanning system (BX63, Olympus, Japan) or via confocal microscopy (FV1000, Olympus, Japan). The images were processed with ImageJ.

### Isolation of single neurons

Three-week-old Ai9 reporter mice were used for rAAV2-retro-Cre-tagBFP injection, while 4-week-old mice were used for RV-∆G-EGFP injection. Single neurons were isolated at the age of 5 weeks, in accordance with the following protocol. First, mice were anesthetized using isoflurane, immediately following which the brains were dissected and placed in fresh ice-cold artificial cerebrospinal fluid (ACSF) containing 124 mM NaCl, 2.5 mM KCl, 1.2 mM NaH_2_PO_4_, 24 mM NaHCO_3_, 5 mM HEPES, 13 mM glucose, 2 mM MgSO_4_, and 2 mM CaCl_2_ (pH: 7.3–7.4). Mouse brains were then sectioned to a thickness of 300 μm in ice-cold ACSF using a vibratome. Tissue samples exhibiting fluorescence were micro-dissected under a fluorescence microscope and cut into small pieces (1–2 mm^2^), following which they were immediately transferred to a tube containing 2 mg/mL papain (LS003120, Worthington, USA) in neurobasal medium (A2477501, Invitrogen, USA) at 35°C for 30 min. To dissociate the cells, the digested tissue pieces were gently triturated using a glass micropipette with a diameter of 50–100 μm under a fluorescence microscope. Fluorescence-labeled neurons were then placed into a new tube using a micropipette as previously described [79]. Finally, approximately 5–20 fluorescence-positive cells from each nucleus were collected into respective tubes containing lysis buffer in order to construct a transcriptome library.

### RNA extraction, RNA-seq library, and single-cell RNA-seq library construction and sequencing

Total RNA was extracted from the dissected tissue using TRIzol™ Reagent (15596026, Invitrogen, USA). The strand-specific sequencing RNA-seq library was constructed in accordance with the manufacturer's protocol using the VAHTS^TM^ Stranded mRNA-seq Library Preparation Kit for Illumina® (NR602-01, Vazyme, China). For single-cell RNA-seq library construction, cDNA was generated in accordance with the Smart-seq2 method [80]. The same amount of cDNA from each sample was used to create library fragments with Tn5, as previously described [81]. The adapter-ligated fragments were amplified using KAPA HiFi DNA polymerase (KK2101, KAPA Biosystems, USA), following which they were purified using AMPure XP beads (1:1 volume ratio with library, A63881, Beckman Coulter, USA). The final library concentrations were assessed using the Qubit dsDNA HS assay (Q32851, Invitrogen, USA) and qPCR. The fragment distribution was determined using high-sensitivity DNA chips on a 2100 Bioanalyzer (G2938C, Agilent Technologies). Sequencing was performed using the 150-bp paired-end configuration on an Illumina HiSeq X-Ten platform.

### Quality control and processing of sequencing data

The datasets generated and analysed during the current study are available in the Gene Expression Omnibus (GEO) repository, https://www.ncbi.nlm.nih.gov/geo/query/acc.cgi?acc=GSE115865. The accession number for the sequencing data reported in this paper is GEO: GSE115865. All raw sequencing data were subjected to a quality check using FastQC. The samples with high-quality reads were collected for downstream analysis. Library construction adapters in reads were trimmed using Trimmomatic, and the clean reads were aligned to the mouse genome GRCm38 (mm10) using HISAT2 [82, 83]. Levels of gene expression were assessed and normalized to fragments per kilobase of transcript per million (FPKM) using StringTie [84]. Differential gene expression analysis was performed using the edgeR package [85]. Kyoto Encyclopedia of Genes and Genomes (KEGG) and Gene Ontology (GO) term enrichment analysis were performed using the cluster Profiler package, as previously described [86]. The t-distributed stochastic neighbor embedding (t-SNE) method implemented in the scikit-learn package was used to reduce the dimension of expression data, as previously described [87]. G2Cdb (http://www.genes2cognition.org/db/Search?text=Mus+musculus) and the Uniprot database (http://www.uniprot.org/uniprot/?query=mouse+membrane&sort=score database) were used to filter membrane and synaptic proteins, respectively. Moreover, a synaptic protein database (EXP) was established in accordance with the experimental data published by Tang et al [88].

### Image analysis

For quantification analysis of virus-labeled regions, brain slices of whole brain in coronal section from each mouse were scanned by the Olympus microscopy slide scanning system. Each brain slice was manually mapped to a corresponding coronal section of the Allen Mouse Brain Atlas via Adobe Photoshop CS5, and brain regions were identified based on DAPI and autofluorescence of the tissue. Fluorescence-positive neurons were manually counted for each brain nucleus. For the semi-quantification of PRV-152, RV-B2C-EGFP, rAAV2-retro-YFP and RV-∆G-EGFP infection, different colors are used to represent different neuron number intervals. For labeling preference analysis of rAAV2-retro-Cre-tagBFP or RV-∆G-EGFP virus-labeled regions upstream from the LHA and MPO, the neuronal number in each region was normalized by the total number of labeled neurons in the whole brain. Data were collected from three mice for each virus.

For the reconstructed image in Fig. 2i, representative brain slices were selected from the PRV-152 and RV-B2C-EGFP injected groups. The fluorescent signal was extracted with the ‘Find Maxima’ function of the ImageJ. Then the signal was reconstructed to the corresponding coronal section according to Allen Mouse Brain Atlas using Adobe Photoshop CS5. Three mice were used for each tracing data.

For immunohistochemistry analysis of cytotoxicity due to virus infection, immunofluorescence-positive oxytocin or vasopressin neurons were manually counted in the paraventricular nucleus on the side of virus injection and compared with the number on the contralateral side. For the signal analysis of Iba1 and GFAP, the mean fluorescence intensity of whole image was measured by Image J. Data were analyzed via unpaired t-tests using GraphPad Prism (version 5.01) software (San Diego, CA). Differences were considered statistically significant when *P* < 0.05. Data were collected from four virus-injected mice and four controls mice for each immunohistochemistry analysis.

## Additional files

Additional file 1: **Figure S1.** Comparison of mono-synaptic labeling between rAAV2-retro and RV-∆G under Cre expression in Ai9 reporter mice. (**A**) Schematic diagram of virus injection into the LHA of Ai9 reporter mice. (**B, C**) Both rAAV2-retro-Cre-tagBFP and RV-∆G-Cre-tagBFP were accurately injected into the LHA. (**D-E’**) Intense labeling was observed in the mPFC (Cg1+PrL) following injection of rAAV2-retro-Cre-tagBFP (D, D’), while weak labeling was observed in the NAc (D, D”) and PVN (E, E’). (**F-G’**) No mPFC labeling was observed following injection of RV-∆G-Cre-tagBFP (F, F’), which was capable of labeling neurons in the NAc (F, F”) and PVN (G, G’). Red, tdTomato expressed by rAAV2-retro-Cre-tagBFP- or RV-∆G-Cre-tagBFP-labeled cells; blue, DAPI. Scale bars = 1 mm for B-G; 200 μm for magnified images. (TIF 4110 kb)

Additional file 2: **Figure S2.** The view of upstream inputs to the LHA by combing rAAV2-retro and RV-∆G labeling results. (**A-E**) The comprehensive upstream circuits of the LHA by combining the data from both rAAV2-retro-Cre-tagBFP and RV-∆G-EGFP labeling in mouse brain were presented as Heat-map on sagittal view. Labeling intensity was quantified by the number of neurons in proportion to the color density. For each upstream nucleus of LHA, the neuron number was quantified by adding rAAV2-retro-Cre-tagBFP- and RV-∆G-EGFP-labeled neurons. (PDF 200 kb)

Additional file 3: **Figure S3.** Complementing TVA receptor in V1 neurons enables EnvA pseudotyped RV infection in layer 5 of V1 cortex. (**A**) Schematic diagram of TVA complementation into V1 cortex to enable EnvA pseudotyped RV infection in the Layer 5 and 6 corticothalamic neurons targeting to dLGN. (**B**) The injection site for EnvA-RV-∆G-DsRed in dLGN. (**C**) Through injection of rAAV9-EGFP-TVA helper virus in V1, TVA receptors were successfully expressed by V1 neurons as indicated by EGFP fluorescence. (**D**) EnvA-RV-∆G-DsRed retrogradely labeled fifth (L5) and sixth layer (L6) of V1 cortex. (**E**) Merged images for C and D. green: rAAV9-EGFP-TVA; red: EnvA-RV-∆G-DsRed; blue, DAPI. Scale bars = 200 μm. (TIF 3920 kb)

Additional file 4: **Figure S4.** Receptor candidates screening and GO analysis on single-cell RNA-seq data of rAAV2-retro-labeled and RV-∆G-labeled neurons. (**A**) Violin plot showed the distribution of gene numbers detected via single-cell RNA sequencing in the rAAV2-retro-Cre-tagBFP and RV-∆G-EGFP groups. (**B**) Flow chart of candidate receptor screening. We first screened for genes commonly expressed in the rAAV2-retro-Cre-tagBFP or RV-∆G-EGFP group, following which the results were filtered using a putative membrane protein database, a putative synaptic protein database, and an experimentally identified synaptic protein database. (**C**) The commonly expressed genes in the rAAV2-retro-Cre-tagBFP group (upper panel) or RV-∆G-EGFP (lower panel) group were filtered using one membrane protein database and two synapse-specific protein databases. (**D-E**) Receptor candidate genes in the rAAV2-retro-Cre-tagBFP (D) and RV-∆G-EGFP groups (E) were classified via GO-term pathway analysis. A pie chart shows the percentage of candidate genes associated with specific categories. The color indicates different pathway. (TIF 6800 kb)

Additional file 5: **Figure S5.** KEGG analysis of differentially expressed genes at the injection site and in retrogradely labeled nuclei. (A-D) KEGG analysis of differential gene expression in the LHA following the injection of rAAV2-retro-YFP (A), in the LHA following RV-∆G-EGFP injection (B), in the region of the mPFC retrogradely labeled by rAAV2-retro-YFP following injection into the LHA (C), and in the region of the NAc retrogradely labeled by RV-∆G-EGFP following injection into the LHA (D). Red, upregulated gene expression; blue, downregulated gene expression. (PDF 478 kb)

Additional file 6: **Figure S6.** RV infection does not influence GFAP and vasopressin expression. (**A-D**) No Iba1 positive signals were detected in contralateral sides of injection site (LHA) and retrogradly labeled sites (mPFC and NAc) by rAAV2-retro-YFP and RV-∆G-EGFP. (**E-L**) Immunostaining signal of astrocyte marker GFAP in the injection site (LHA) and retrogradely labeled sites (mPFC and NAc) by viral tracer (rAAV2-retro-YFP or RV-∆G-EGFP) and Mock control. (**M**) Quantification of mean fluorescence intensity (Mean±SEM) of GFAP in LHA after virus injection. rAAV2-retro-YFP: 10.90±0.7199, Mock:11.07±1.399, rAAV2-retro-YFP vs Mock: P=0.9173; RV-∆G-EGFP: 9.804±1.297, Mock: 8.374±0.8445, RV-∆G-EGFP vs Mock: P=0.3912; *n* = 4, n is mice number. (**N**) Quantification of mean fluorescence intensity (Mean±SEM) of GFAP in mPFC injected with rAAV2-retro-YFP and PBS. rAAV2-retro-YFP: 7.397±1.374, PBS: 7.204±0.7184, P=0.9048, *n* = 4. (**O**) Quantification of mean fluorescence intensity (Mean±SEM) of GFAP in NAc injected with RV-∆G-EGFP injected and PBS. RV-∆G-EGFP: 8.104±0.8872, Mock: 10.53±1.049, P=0.1276; *n* = 4, n is mice number. (**P**) Immunostaining for oxytocin in PVN neurons 14 days following the injection of RV-∆G-EGFP into the LHA. (**Q-R**) Boxed areas in P were magnified to show oxytocin-positive neurons in the ipsilateral and contralateral PVN to the virus injection side. (**S**) Higher-magnification images of the right dashed box depicting the ipsilateral PVN in R with red color and merged color. Green, GFP expressed by RV-∆G-EGFP; red, oxytocin positive signal. (**T**) Quantification of oxytocin-positive neurons number (Mean±SEM) in the ipsilateral and contralateral PVN to the virus injection side. Contra: 21.25±1.931, Ipsi: 52.50±18.150, P=0.0097, *n* = 4, n is mice number. (**U**) Immunostaining for vasopressin in PVN neurons 7 days following the injection of RV-∆G-EGFP into the LHA. (**V-W**) Boxed areas in U were magnified to show vasopressin-positive neurons in the ipsilateral and contralateral PVN to the virus injection side. (**X**) Higher-magnification images of the dashed box depicting the ipsilateral PVN in U with red color and merged color. Green, GFP expressed by RV-∆G-EGFP; red, vasopressin positive signal. (**Y)** Quantification of vasopressin-positive neurons (Mean±SEM) in the ipsilateral and contralateral PVN, Contra:49.00±6.258, Ipsi:54.00±5.370, P=0.5665, *n* = 4, n is mice number. Scale bars = 100 μm for A-L; 200 μm for P and U; 50 μm for Q-S and V-X. MFI: mean fluorescence intensity. Unpaired t-tests, **P<0.01. (TIF 7730 kb)

## Abbreviations

CAV: Canine adenovirus
CNS: Central nervous system
Cre: Cre recombinase
dLGN: Dorsal lateral geniculate nucleus
EnvA: Avian sarcoma leukosis virus envelope protein
GEO: Gene Expression Omnibus
GO-term: Gene Ontology Term
HSV: Herpes simplex virus
KEGG analysis: Kyoto Encyclopedia of Genes and Genomes
LHA: Lateral hypothalamic area
MPO: Medial preoptic nucleus
PRV: Pseudorabies virus
rAAV2-Retro: retro adeno-associated virus
RV: Rabies virus
RV-∆G: Glycoprotein-deleted rabies virus
TK: Thymidine kinase
TVA: Avian sarcoma leukosis virus receptor
VSV: Vesicular stomatitis virus
