# Supplementary figures and images for: Differences in neurotropism and neurotoxicity among retrograde viral tracers
Source: Mol Neurodegener. 2019 Feb 8;14:8. doi: 10.1186/s13024-019-0308-6 (PMC6368820; doi:10.1186/s13024-019-0308-6)

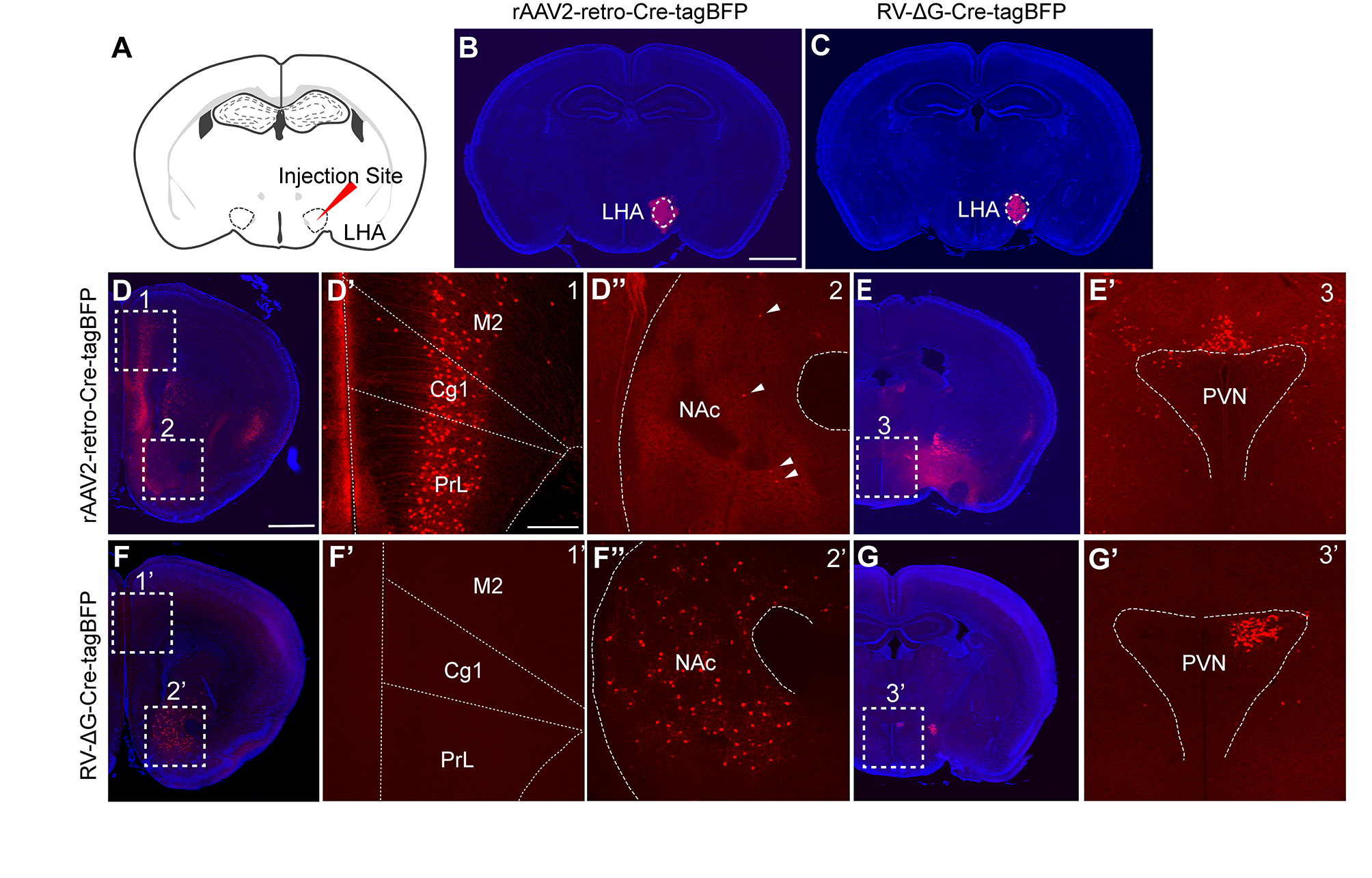

Supplement: Supplementary file 1 — Figure S1. Comparison of mono-synaptic labeling between rAAV2-retro and RV-∆G under Cre expression in Ai9 reporter mice. (A) Schematic diagram of virus injection into the LHA of Ai9 reporter mice. (B, C) Both rAAV2-retro-Cre-tagBFP and RV-∆G-Cre-tagBFP were accurately injected into the LHA. (D-E’) Intense labeling was observed in the mPFC (Cg1+PrL) following injection of rAAV2-retro-Cre-tagBFP (D, D’), while weak labeling was observed in the NAc (D, D”) and PVN (E, E’). (F-G’) No mPFC labeling was observed following injection of RV-∆G-Cre-tagBFP (F, F’), which was capable of labeling neurons in the NAc (F, F”) and PVN (G, G’). Red, tdTomato expressed by rAAV2-retro-Cre-tagBFP- or RV-∆G-Cre-tagBFP-labeled cells; blue, DAPI. Scale bars = 1 mm for B-G; 200 μm for magnified images. (TIF 4110 kb) [file 13024_2019_308_MOESM1_ESM.tif]

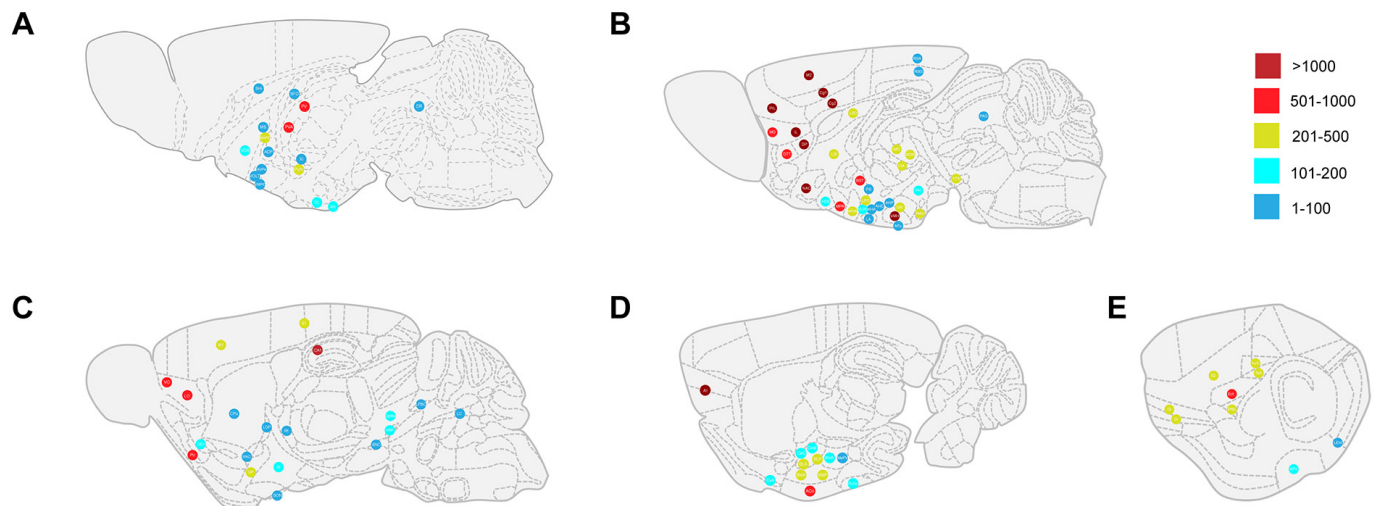

Supplement: Supplementary file 2 — Figure S2. The view of upstream inputs to the LHA by combing rAAV2-retro and RV-∆G labeling results. (A-E) The comprehensive upstream circuits of the LHA by combining the data from both rAAV2-retro-Cre-tagBFP and RV-∆G-EGFP labeling in mouse brain were presented as Heat-map on sagittal view. Labeling intensity was quantified by the number of neurons in proportion to the color density. For each upstream nucleus of LHA, the neuron number was quantified by adding rAAV2-retro-Cre-tagBFP- and RV-∆G-EGFP-labeled neurons. (PDF 200 kb) [file 13024_2019_308_MOESM2_ESM.pdf]

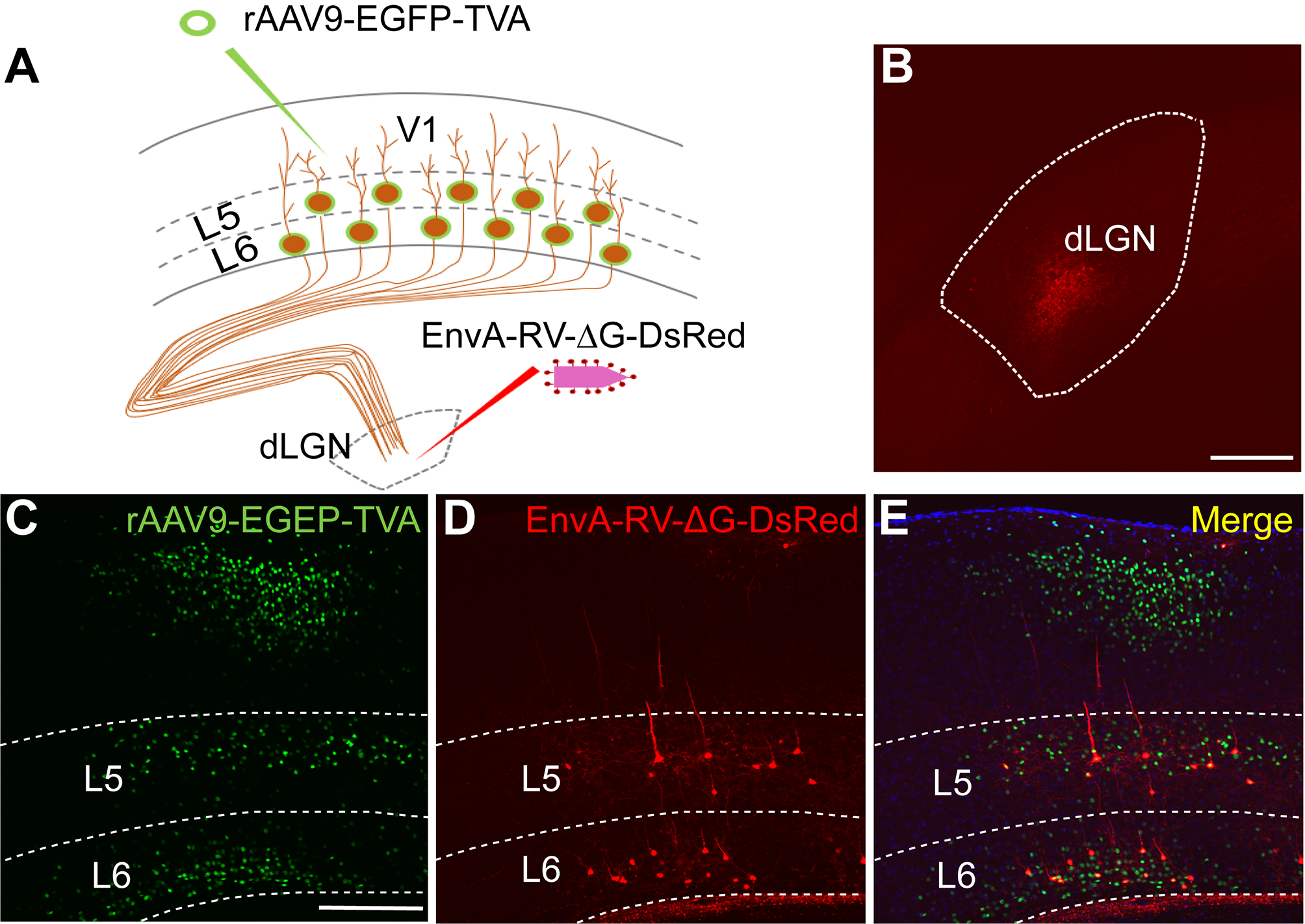

Supplement: Supplementary file 3 — Figure S3. Complementing TVA receptor in V1 neurons enables EnvA pseudotyped RV infection in layer 5 of V1 cortex. (A) Schematic diagram of TVA complementation into V1 cortex to enable EnvA pseudotyped RV infection in the Layer 5 and 6 corticothalamic neurons targeting to dLGN. (B) The injection site for EnvA-RV-∆G-DsRed in dLGN. (C) Through injection of rAAV9-EGFP-TVA helper virus in V1, TVA receptors were successfully expressed by V1 neurons as indicated by EGFP fluorescence. (D) EnvA-RV-∆G-DsRed retrogradely labeled fifth (L5) and sixth layer (L6) of V1 cortex. (E) Merged images for C and D. green: rAAV9-EGFP-TVA; red: EnvA-RV-∆G-DsRed; blue, DAPI. Scale bars = 200 μm. (TIF 3920 kb) [file 13024_2019_308_MOESM3_ESM.tif]

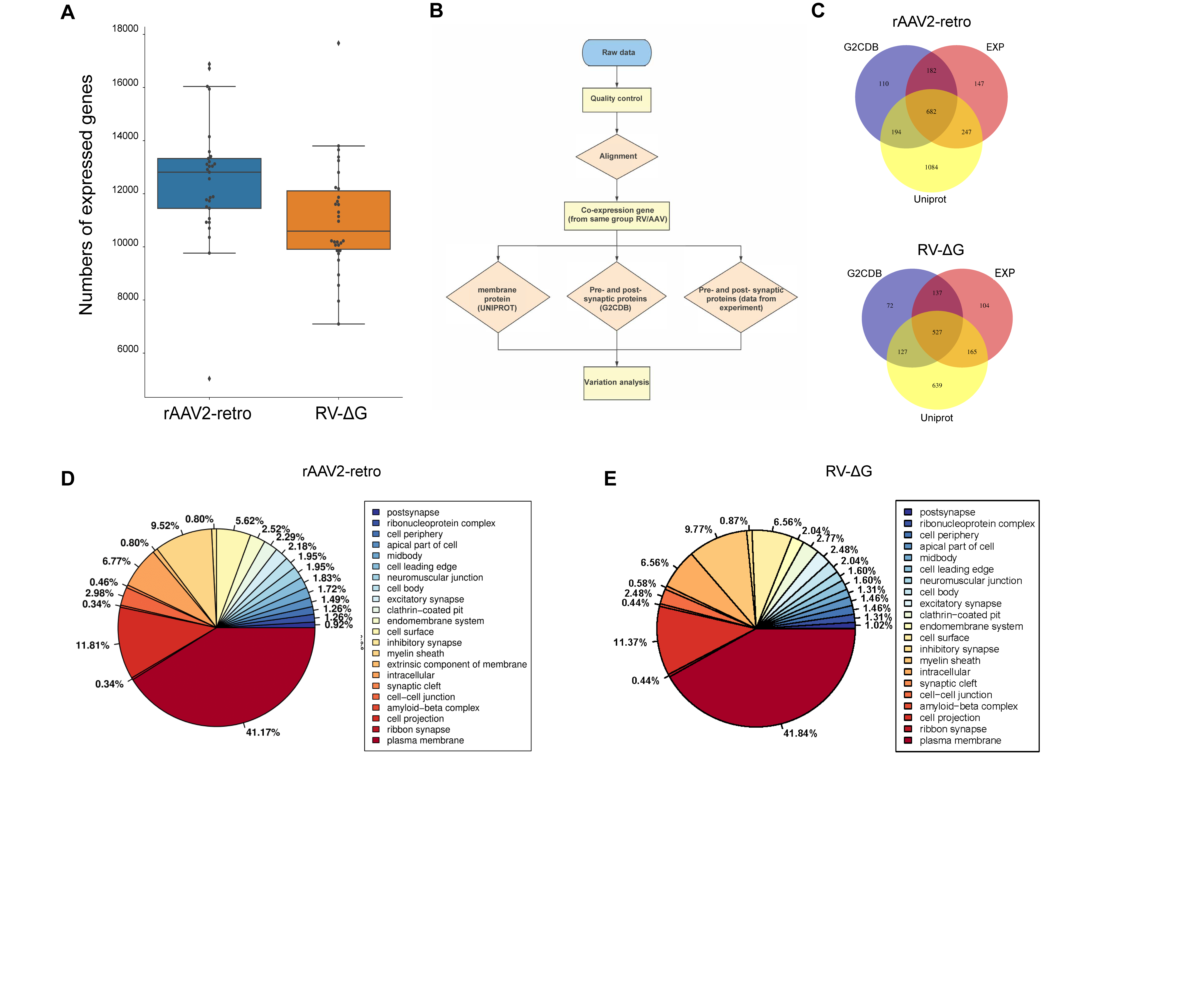

Supplement: Supplementary file 4 — Figure S4. Receptor candidates screening and GO analysis on single-cell RNA-seq data of rAAV2-retro-labeled and RV-∆G-labeled neurons. (A) Violin plot showed the distribution of gene numbers detected via single-cell RNA sequencing in the rAAV2-retro-Cre-tagBFP and RV-∆G-EGFP groups. (B) Flow chart of candidate receptor screening. We first screened for genes commonly expressed in the rAAV2-retro-Cre-tagBFP or RV-∆G-EGFP group, following which the results were filtered using a putative membrane protein database, a putative synaptic protein database, and an experimentally identified synaptic protein database. (C) The commonly expressed genes in the rAAV2-retro-Cre-tagBFP group (upper panel) or RV-∆G-EGFP (lower panel) group were filtered using one membrane protein database and two synapse-specific protein databases. (D-E) Receptor candidate genes in the rAAV2-retro-Cre-tagBFP (D) and RV-∆G-EGFP groups (E) were classified via GO-term pathway analysis. A pie chart shows the percentage of candidate genes associated with specific categories. The color indicates different pathway. (TIF 6800 kb) [file 13024_2019_308_MOESM4_ESM.tif]

A

## LHA: rAAV2-retro

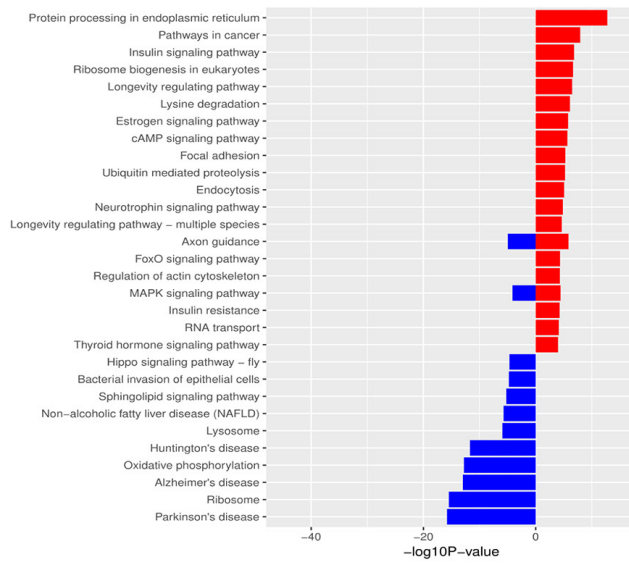

B

## LHA: RV-ΔG

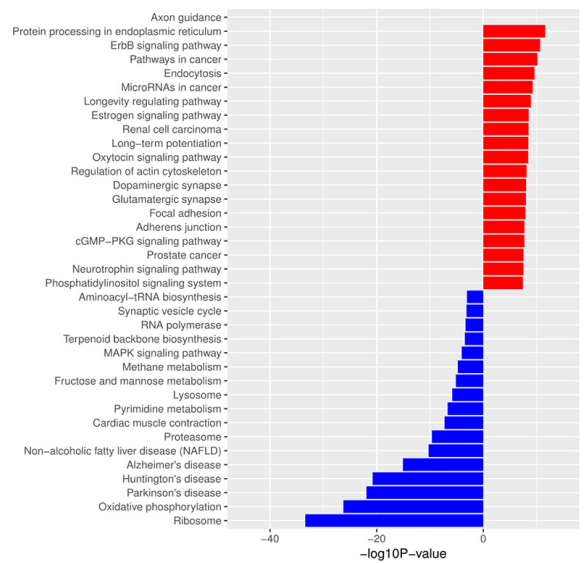

C

## mPFC: rAAV2-retro

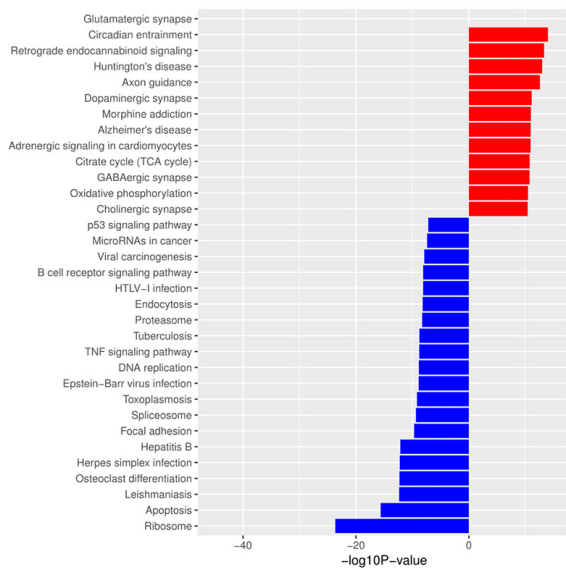

D

## NAc: RV-ΔG

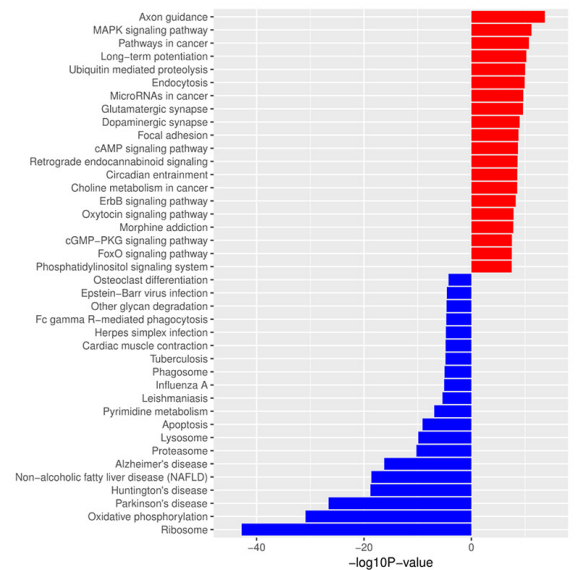

Supplement: Supplementary file 5 — Figure S5. KEGG analysis of differentially expressed genes at the injection site and in retrogradely labeled nuclei. (A-D) KEGG analysis of differential gene expression in the LHA following the injection of rAAV2-retro-YFP (A), in the LHA following RV-∆G-EGFP injection (B), in the region of the mPFC retrogradely labeled by rAAV2-retro-YFP following injection into the LHA (C), and in the region of the NAc retrogradely labeled by RV-∆G-EGFP following injection into the LHA (D). Red, upregulated gene expression; blue, downregulated gene expression. (PDF 478 kb) [file 13024_2019_308_MOESM5_ESM.pdf]

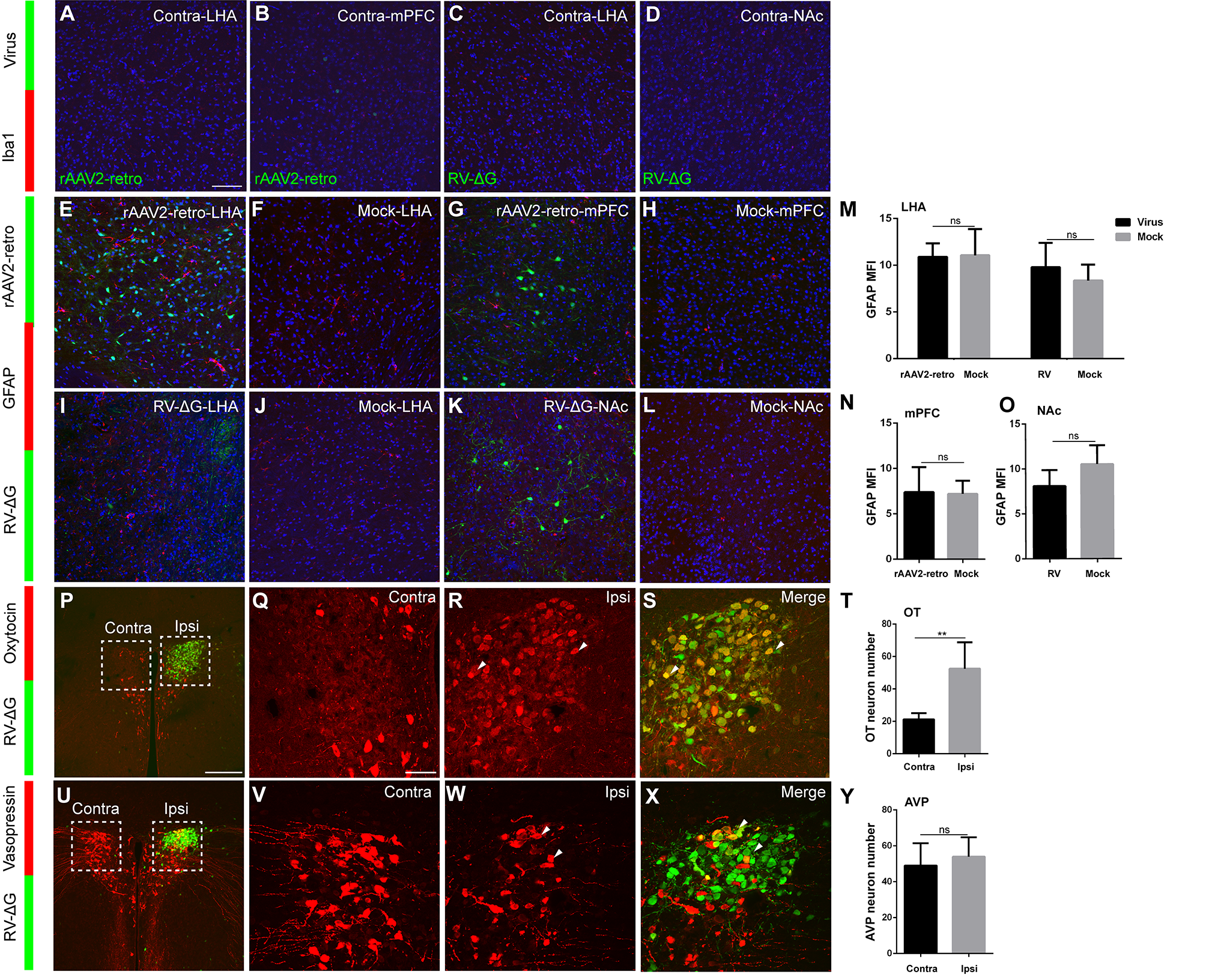

Supplement: Supplementary file 6 — Figure S6. RV infection does not influence GFAP and vasopressin expression. (A-D) No Iba1 positive signals were detected in contralateral sides of injection site (LHA) and retrogradly labeled sites (mPFC and NAc) by rAAV2-retro-YFP and RV-∆G-EGFP. (E-L) Immunostaining signal of astrocyte marker GFAP in the injection site (LHA) and retrogradely labeled sites (mPFC and NAc) by viral tracer (rAAV2-retro-YFP or RV-∆G-EGFP) and Mock control. (M) Quantification of mean fluorescence intensity (Mean±SEM) of GFAP in LHA after virus injection. rAAV2-retro-YFP: 10.90±0.7199, Mock:11.07±1.399, rAAV2-retro-YFP vs Mock: P=0.9173; RV-∆G-EGFP: 9.804±1.297, Mock: 8.374±0.8445, RV-∆G-EGFP vs Mock: P=0.3912; n = 4, n is mice number. (N) Quantification of mean fluorescence intensity (Mean±SEM) of GFAP in mPFC injected with rAAV2-retro-YFP and PBS. rAAV2-retro-YFP: 7.397±1.374, PBS: 7.204±0.7184, P=0.9048, n = 4. (O) Quantification of mean fluorescence intensity (Mean±SEM) of GFAP in NAc injected with RV-∆G-EGFP injected and PBS. RV-∆G-EGFP: 8.104±0.8872, Mock: 10.53±1.049, P=0.1276; n = 4, n is mice number. (P) Immunostaining for oxytocin in PVN neurons 14 days following the injection of RV-∆G-EGFP into the LHA. (Q-R) Boxed areas in P were magnified to show oxytocin-positive neurons in the ipsilateral and contralateral PVN to the virus injection side. (S) Higher-magnification images of the right dashed box depicting the ipsilateral PVN in R with red color and merged color. Green, GFP expressed by RV-∆G-EGFP; red, oxytocin positive signal. (T) Quantification of oxytocin-positive neurons number (Mean±SEM) in the ipsilateral and contralateral PVN to the virus injection side. Contra: 21.25±1.931, Ipsi: 52.50±18.150, P=0.0097, n = 4, n is mice number. (U) Immunostaining for vasopressin in PVN neurons 7 days following the injection of RV-∆G-EGFP into the LHA. (V-W) Boxed areas in U were magnified to show vasopressin-positive neurons in the ipsilateral and contralateral PVN to the virus injec [file 13024_2019_308_MOESM6_ESM.tif]
